# Adapted hepatitis C virus clone infects innate immunity-deficient mouse hepatocytes with minimal human HCV entry factors

**DOI:** 10.1016/j.jhepr.2025.101328

**Published:** 2025-01-18

**Authors:** Julie Ann Sheldon, Melina Winkler, Qinggong Yuan, Nicola Frericks, Richard John Phillip Brown, Csaba Miskey, Natascha Gödecke, Sara Behme, Katharina Rox, Giorgia Mysegades, Florian Vondran, Dagmar Wirth, Thomas Pietschmann

**Affiliations:** 1 Institute for Experimental Virology, TWINCORE, Centre for Experimental and Clinical Infection Research, a joint venture Between the Helmholtz Centre for Infection Research and the Hannover Medical School, Hannover, Germany; 2 Department of Gastroenterology, Hepatology, Infectious Diseases and Endocrinology, Hannover Medical School, Hannover, Germany; 3 Department for Molecular and Medical Virology, Ruhr-University Bochum, Bochum, Germany; 4 Division of Veterinary Medicine, Paul Ehrlich Institute, Langen, Germany; 5 Division of Medical Biotechnology, Paul Ehrlich Institute, Langen, Germany; 6 Model Systems for Infection and Immunity, Helmholtz Centre for Infection Research, Braunschweig, Germany; 7 Department of Chemical Biology, Helmholtz Centre for Infection Research, Braunschweig, Germany; 8 Cluster of Excellence RESIST (EXC 2155), Hannover Medical School, Hannover, Germany; 9 Department of Virology, Hannover Medical School, Hannover, Germany; 10 Department for General, Visceral and Transplant Surgery, Hannover Medical School, Hannover, Germany; 11 Clinic for General, Visceral and Transplant Surgery, University Hospital Rheinisch-Westfälische Technische Hochschule Aachen, Aachen, Germany; 12 Institute of Experimental Hematology, Hannover Medical School, Hannover, Germany; 13 German Center for Infection Research (DZIF), Partner Site Hannover-Braunschweig, Hannover, Germany

**Keywords:** Adaptation, Species tropism, Replication, Molecular clone

## Abstract

**Background & Aims:**

Hepatitis C virus (HCV) has a narrow species tropism and cannot infect mice. To understand HCV species tropism and to develop better animal models, we adapted HCV to infect mouse cells deficient in innate immunity and with minimal human HCV host factors.

**Methods:**

HCV was adapted via passaging an HCV infectious virus clone several times in human hepatoma cells, mouse liver cells, and eventually primary mouse hepatocytes deficient in innate immunity and ectopically expressing human occludin and human CD81. Using RNAseq the sequence of the adapted virus was analyzed, and several clones were generated to study replication and infection kinetics as well as neutralization assays in several human/mouse cell lines and primary hepatocytes from human, mouse, and macaques.

**Results:**

Accumulation of 35 non-synonymous and 66 synonymous mutations correlated with >1,000-fold enhanced production of infectious progeny from primary mouse hepatocytes. These mutations did not confer drug resistance or evasion from innate immunity. They did not enhance fitness in human or macaque hepatocytes. We show that non-synonymous mutations are necessary and sufficient for adaptation, and that changes to the glycoproteins are not essential. Mutations outside of viral envelope proteins enhanced specific infectivity and facilitated viral spread in murine cells.

**Conclusions:**

This study reveals key viral factors governing HCV species tropism. The mouse-adapted HCV opens up possibilities for the development of animal models to analyze HCV pathogenesis, immune control, and vaccine development.

**Impact and implications:**

This work demonstrates the feasibility in principle of HCV adaptation to replication in and infection of non-human cells. This is made possible by a manageable number of non-synonymous mutations and opens up new ways to elucidate the principles of HCV species tropism and to develop important animal models for HCV research in the long term.

## Highlights

• Mouse-adapted HCV efficiently infects primary mouse hepatocytes deficient in innate immune signaling.
• Non-synonymous mutations in structural and non-structural proteins confer adaptation.
• Envelope protein mutations contribute to adaptation but are not essential.
• A mouse-adapted cell clone with parental envelope proteins infected mouse hepatocytes *ex vivo.*
• Enhanced RNA replication and increased release of more infectious particles confer mouse adaptation.

## Graphical abstract

**Figure:**
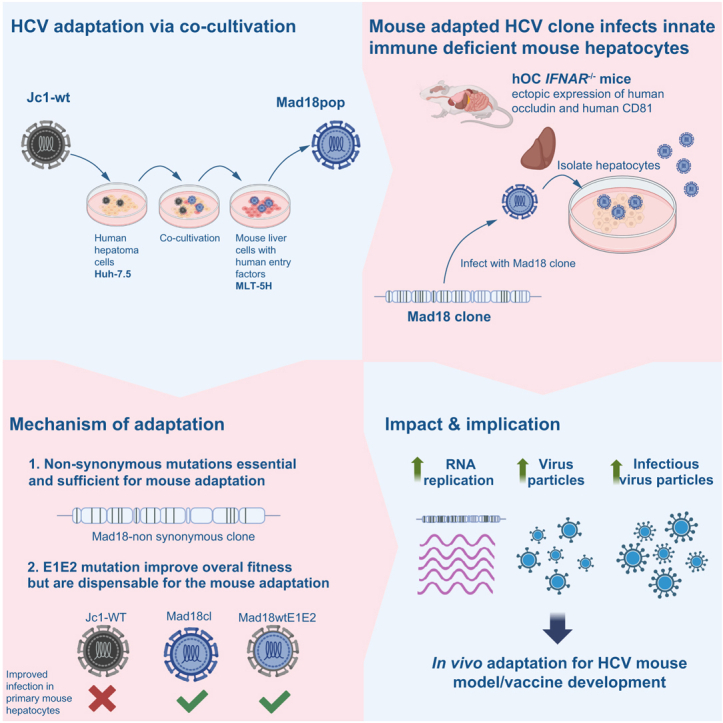

## Introduction

The World Health Organization estimates that 50 million people are chronically infected with hepatitis C virus (HCV) and within 20 years, 15–30% are at risk of cirrhosis. Although pan-genotypic direct-acting antivirals can cure HCV infections, treatment remains expensive and access is limited (Hepatitis C). More recently, the US has seen an increase in HCV infections as a result of the opioid crisis. (Deeper Look: Opioids - HepVu). Moreover, treatment induced-cure does not protect from viral reinfection and research suggests that the incidence of reinfection is rising, particularly in prison settings^1^ or among people who inject drugs (PWID).^2^ Consequently, HCV transmission remains high. Based on empirical data and treatment forecasts, the number of new chronic infections is estimated to remain at an average of 1.42 million every year until 2030.^3^ A prophylactic vaccine would aid HCV control if applied to groups at risk for transmissions such as people who inject drugs (PWID), men who have sex with men (MSM), prisoners, sex workers, healthcare workers and populations in areas with high HCV seroprevalence, however until now, no vaccine is available. The development of an HCV vaccine is hampered by the lack of immunocompetent animal models, as no efficacy studies of vaccine candidates in preclinical animal models are possible. One of the main challenges for small animal model development is the narrow host specific tropism of HCV, which only efficiently infects humans and chimpanzees. Much effort has been invested in the development of genetically modified mice that are susceptible to HCV because of the expression of the human HCV host factors. This was enabled by discovery of four key HCV cell entry factors (scavenger receptor class B type 1 [SR-B1],^4^ CD81, occludin [OCLN], and claudin-1 [CLDN1]^5^ of which CD81^6^ and OCLN^7^ are used in a species-specific fashion.^7^^,^^8^ However, even in receptor transgenic mice with ablated innate immune signaling, HCV replication levels remain minimal,^9^ suggesting that lack of other human factors limits infection. With the recent discovery of several novel hepaciviruses, in particular in a broad range of rodents[10], [11], [12], [13], [14], [15] coupled with the increasing number of viruses that have jumped the species barrier, it is becoming more relevant to understand the mechanisms underlying interspecies transmission.

In this project we took advantage of the error-prone RNA-dependent RNA polymerase of HCV and high levels of virus production in tissue culture. As with all RNA viruses, HCV forms a cloud of related genetic variants, also termed as a quasispecies. In a stable environment the consensus sequence in a quasispecies remains the same; however, upon entering a new environment a bottleneck is created, and the viral population rapidly adapts to novel selective forces, changing the consensus sequence.^16^ We gradually and extensively adapted an HCV population to replicate efficiently in mouse liver cells without losing infectivity to human cells. First, by co-cultivation of both human and mouse hepatic cells and eventually passaging in primary mouse hepatocytes. We further characterized this adaptation towards mouse liver cell tropism by creating molecular clones harboring the consensus mutations. Finally, we pinpointed the impact of the adaptation on distinct stages of the viral life cycle, which ultimately enables increased replication and infection of primary mouse hepatocytes and thereby cross-species transmission.

## Materials and methods

### Cell lines

Huh-7.5,^17^ Lunet N#3,^18^ and MLT-5H (miR-122, MLT-MAVS^-/-^ miR-122 hhhhh)^19^ cell lines were cultured in Dulbecco’s modified Eagle’s medium (Invitrogen, Darmstadt, Germany) complete, which was supplemented with 1% (v/v) non-essential amino acids (Invitrogen), 2 mM L-glutamine (Invitrogen), 1% (v/v) penicillin/streptomycin (Thermo Fisher Scientific, Darmstadt, Germany) and 10% fetal calf serum (FCS) (PAA Laboratories GmbH, Cölbe, Germany) (DMEM complete). MLT-5H were further supplemented with 5 μg/ml blasticidin (Invitrogen), 5 μg/ml puromycin (Sigma-Aldrich, Taufkirchen, Germany) and 750 μg/ml G418 (Biochrom, Berlin, Germany) for the selection of the ectopically expressed, human occludin, human claudin 1, human CD81, human ApoB and human Scavenger receptor B1.^19^

Primary human and mouse hepatocytes were cultured in HCM media (Lonza, Cologne, Germany). Ruxolitinib (Rux) at a final concentration of 10 μM (Adipogen/Biomol, Hamburg, Germany) was added – when indicated – 16 h before infection and at every media change (24–48 h).^20^ Telaprevir (Selleckchem, Cologne, Germany) was added at a final concentration of 1 μM when indicated 4 h post infection/electroporation.

### Viruses, replicons, and cloning

Jc1-WT (plasmid pFK-JFH1/J6/C-846),^21^ P100pop (provided as a kind gift from Esteban Domingo, Madrid, Spain), and subsequent viral populations and viral clones were produced by electroporation (clones) as described by Kato *et al.*,^22^ or infection (populations) of Huh-7.5 cells at an approximate multiplicity of infection (MOI) of 1. For both electroporated and infected cells, supernatants were subsequently collected at 60, 72, and 80 h post infection/electroporation and filtered through a 0.45 μM cellulose acetate filter (Millipore, Darmstadt, Germany). Virus progeny was then pooled and concentrated using Amicon 100-kDa spin columns (Millipore) or an Amicon stirred cell with a 100-kDa filter (Millipore).

The Mad18 and P100 clones Mad18cl, Mad18ns, and P100cl^23^ were synthesized by Genscript (Genscript, Rijswijk, Netherlands) inserted in the pUC57 vector based on the Mad18pop and P100pop consensus sequences. The FLAG-tag and signal sequence (originally inserted after the luciferase transgene and upstream of NS2), were omitted in the clones to generate authentic HCV genomes without foreign gene sequences. For the generation of the Mad18WTE1E2 and P100WTE1E2 clones, Mad18cl or P100cl were digested with *NcoI* and *NotI* and replaced by a synthetic fragment (Genscript) spanning the same region and only containing Mad18clone’s adaptive mutations outside the glycoprotein-coding regions.

The Mad18ns subgenomic replicon (SGR) was inserted into the JFH1 SGR (pFK-i389LucNS3-3′)^24^ using restriction enzymes *MreI* and *BbvCI*.

The two mutations in core (P138A and P143A) were introduced into Jc1-WT, Mad18cl, and Mad18WTE1E2 using restriction enzymes *Spf*1 (Jc1) or *Nco*1 (mad18cl and Mad18WTE1E2) and *BsiWI* and the synthesized fragment (Genscript) containing to the two mutations.

### *In vitro* transcription

HCV full-length genome or subgenome replicons were linearized with either *Mlu* 1 (Jc1-wt, Jc1mCD81, JFH1-SGR, or Mad18ns-SGR) or *Xba* 1 (Mad18cl, Mad18ns, Mad18WTE1E2, P100cl, or P100WTE1E2 followed by purification using Qiaprep Spin Miniprep Kit [Qiagen, Hilden, Germany]). Linearized plasmid DNA was then *in vitro* transcribed in RRL buffer (0.2 M HEPES, pH 7.5, 0.2 M MgCl_2_, 0.2 M spermidine, 0.2 M DTT) containing ribonucleotide tri-phosphates (rNTPs) (3 mM each rNTP), 100 U RNasin ribonuclease inhibitor (Promega, Walldorf, Germany) and 60 U T7 polymerase (Promega) for 2 h at 37 °C then boosted with 30 U T7 polymerase for further 2 h. Finally, the *in vitro* transcription reaction was terminated by addition of 7.5 U RQ1 Dnase (Promega) to the reaction mix and incubated for 30 min at 37 °C. The cRNA was purified using the NucleoSpin RNA Clean-up kit (Macherey-Nagel, Düren, Germany). Quality and quantity of the *in vitro* transcripts were checked by agarose gel electrophoresis and a Nanodrop One (Thermo Scientific).

### Mice and plating primary hepatocytes

hOC^hep^ mice with hepatocyte-specific expression of human occludin and human CD81 (hOC^hep^ hOC*LN*^-/-^ h*CD81*^-/-^) were generated by breeding conditional hOC^con^ mice^25^ with AlbCre mice and selected for homozygosity of the human entry factors. Breeding of hOC^hep^ mice to IFNAR^-/-^ (interferon A receptor) mice generated by hOC^hep^ IFNAR^-/-^ mice.

Hepatocytes were isolated from mice as previously described^26^ with the approval of the ethical board of Lower Saxony state office for consumer protection and food safety (LAVES, Oldenburg Germany) under the animal testing application 21-3745. Briefly, following anesthesia with Ketamin (Albrecht, Aulendorf, Germany) and Rompun (Bayer, Leverkusen, Germany), a catheter was inserted into the hepatic portal vein, connected to a flow pump and the *vena cava* was cut. The liver was perfused with pre-warmed Earle’s Balanced Salt Solution (EBSS) (Gibco, Darmstadt, Germany) solution containing 0.5 mM ethylene 28 glycol tetra-acetic acid (EGTA) (Sigma-Aldrich) and 10 mM HEPES buffer (Sigma-Aldrich). Subsequently, EBSS supplemented with 10 mM HEPES buffer and 100 μg/ml Liberase (Roche, Basel, Switzerland) was applied to the liver for enzymatic digestion at 37 °C. After 10–12 min, the liver was carefully disconnected, and the tissue manually disrupted with sterile scissors and scalpel in DMEM containing 10% FCS. The suspended hepatocytes were passed through a 100 μm nylon filter and then centrifuged twice at 50 × *g* for 5 min at 4 °C. Cell pellets were resuspended in complete DMEM medium. Cell viability was tested using Trypan Blue (Gibco) before plating on collagen (Roche) coated six-well dishes at a density of 5 × 10^5^ cells per well. Two hours after plating, cells were maintained in supplemented HCM media (Lonza).

Primary human hepatocytes were isolated from liver tissue from donors undergoing partial hepatectomy and were obtained with written informed consent approved by the Ethics Commission of Hannover Medical School (Ethik-Kommission der MHH, no. 252-2008).

Three primary macaque hepatocytes isolates were purchased from Lonza (# RHCP01), thawing and plating were as the manufacturer’s instructions. Briefly, cells were thawed and directly transferred to 50 ml of thawing media (#MCRT50, Lonza). The cells were then centrifuged 50 × *g* for 8 min at room temperature and carefully resuspended in plating media (#MP250, Lonza) followed by plating in 24-well plates at a density of 3.75 × 10^4^ cells per well, after 6 h, media was replaced with HCM media (Lonza) ± Rux (10 μM).

### *In vitro* and *ex vivo* infections

Cells were seeded to semi-confluency in 24-well plates (Huh-7.5, MLT-5H, primary human hepatocytes [PHHs], primary hepatocytes from macaques [PMacHs]). Sixteen hours before infection, Rux (final concentration 10 μM) was supplemented when indicated; infections were normalized to HCV copies numbers. Five hours after infection, cells were washed five times with PBS and replaced with fresh media containing Rux and/or 5 μM telaprevir (Selleckchem, Cologne, Germany) when required.

### *In vitro* transfections

Cells were electroporated with replicon or full-length viral transcripts as previously described,^22^ then subsequently seeded to semiconfluency in 24-well plates, 4 h post electroporation cells were washed with PBS then treated with either DMSO or 1 μM telaprevir (TPV) in DMEM complete. Cell lysates were collected at 4, 24, 48, and 72 h post electroporation for subsequent RNA extraction and quantification.

### RNA extraction and quantification

Intracellular RNA from cell lysates was extracted using the Macherey-Nagel NucleoSpin RNA Clean-up kit according to the manufacturers’ instructions. Extracellular RNA from the supernatant was extracted using Zymo RNA viral kit (Zymo Research, Freiburg, Germany) according to the manufacturers’ instructions. Subsequently, HCV RNA was quantified by RT-qPCR using the LightCycler 480 RNA Master Hydrolysis Probes Kit (Roche) and primers 5′-TCTGCGGAACCGGTGAGTA-3′ and 5′-GGGCATAGAGTGGGTTTATCCA-3′ and probe 5′-[6FAM]-AAAGGACCCAGTCTTCCCGGCAA-[TMR]-3′ according to the manufacturers’ instructions. The PCR reaction protocol was set as follows: 63 °C for 3 min, 95 °C for 30 s, and 45 cycles with each 95 °C for 15 s and 60 °C for 30 s, before a final cooling 40 °C for 30 s was performed. The HCV RNA copies were determined using serially diluted *in vitro* transcribed HCV full-length RNA with a known copy number as an internal standard, followed by normalization to the total RNA of each sample. Technical duplicates were performed for each sample.

### Immunofluorescence

After infection, cells were fixed for 20 min with either 95% ethanol and 5% acetic acid for primary mouse hepatocytes (PMHs) or 3% paraformaldehyde for the MLT-5H and Huh7.5 cells, followed by a 10-min permeabilization in 0.1% Triton-X100/PBS. Cells were then washed three times in PBS and the HCV NS5A was detected by incubating the cells for 1 h with a NS5A monoclonal antibody (clone 9E10, Cell Essentials, Boston, MA, USA) in 5% goat serum/PBS, followed by a further three PBS washes and a 1-h incubation with fluorophore-conjugated anti-mouse (Alexa Fluor 488 rabbit anti-mouse immunoglobulin G). After three PBS washes, nuclei staining was performed using a 0.5 μg/ml DAPI solution in H_2_O. Cells were observed using a fluorescent microscope (Olympus XI81, Hamburg, Germany) or quantified using an Immunospot analyzer (Cellular Technology Limited, Rutesheim, Germany).

### Western blot

PMHs were infected with 100 HCV RNA copies/cell, the cells were washed five times with PBS, 5 h post infection. For a conditioned media control, cells were mock infected with supernatant of uninfected cells 72 h post seeding. At 96 h.p.i. cells were washed once with PBS before lysis with 50 mM Tris–HCl pH 7.4, 150 mM NaCl, 1% Triton X-100, 60 mM beta-glycerol phosphate, 15 mM 4-nitrophenylphosphate, 1 mM sodium orthovanadate, 1 mM sodium fluoride, Pierce protease inhibitor mix. Cell lysates were cleared by centrifugation at 16,000 × *g* for 10 min at 4 °C before total protein quantification using the Qubit 4 Fluorometer (Thermo Fisher). Twelve micrograms of protein were mixed with sample buffer (75 mM Tris–HCL pH 6.8, 4% SDS, 40% glycerol, 200 mM DTT, 0.005% Bromophenol Blue) and heated at 95 °C for 5 min before protein separation by SDS-PAGE was performed using NuPAGE 4–12% bis-tris gels (Thermo Fisher Scientific) in MES buffer (50 mM 2-(*N*-morpholino) ethanesulfonic acid, 50 mM Tris–HCL, 0.1% SDS, 1 mM EDTA). Proteins were transferred to PVDF membranes using the BioRad Turboblot machine. Membranes were blocked with 5% bovine serum albumin in TBS-Tween 0.1% for 1 h at room temperature. Subsequently, overnight incubation at 4 °C of the primary antibodies, non-purified mouse anti-Core clone C7-50 (1:200, kindly provided Jean Dubuisson) and rabbit anti-GAPDH (1:1,000, Sigma-Aldrich #G9545) was performed. Membranes were washed three times for 10 min with TBS-Tween 0.1% before horseradish peroxidase-conjugated secondary antibodies (Goat anti-mouse HRP IgG [Sigma-Aldrich # A4416], Goat anti-rabbit HRP IgG [Jackson Immuno Research #111035003, Ely, UK], 1:10,000) were applied for 1 h at room temperature. Membranes were again washed three times for 10 min with TBS-Tween 0.1% before protein expression was detected using the Amersham ECL Prime kit (Cytiva, Marlborough, MA, USA) and the ChemoStar Professional Imager (Isogen Life Science, De Meern, Netherlands).

### Limiting dilution assay

Infectious virus release was titrated on Huh-7.5 cells, Lunet N#3 hCD81 or Lunet N#3 mCD81 using the TCID_50_ assay as previously described^27^ and calculated using the Spearman and Kärber method.^28^

### Illumina sequencing of virus populations

Viral RNA isolated using the Macherey-Nagel NucleoSpin RNA Clean-up kit was prepared using a modified protocol of the NNSR priming method^29^ and sequenced on a MiSeq instrument with a paired-end 2 × 300 setting as previously described.^26^

### Sequence analysis

The MiSeq RNAseq data was analyzed using CLC Genomics Workbench (Qiagen). Raw FASTQ files were trimmed, realigned and mapped against the Jc1-SP consensus sequence (accession number OQ726017), with a 5% error cut-off. Mega 6^30^ and SnapGene V7.02 (Dotmatics, Boston, MA, USA) software were further used to align and map the consensus sequences from each viral population.

### Neutralization and receptor blocking assays

Huh-7.5 cells were seeded in poly-L-lysine-coated 96-wells at a density of 2 × 10^4^ cells/well. Two days after seeding, cells (receptor blocking) were incubated with a serial dilutions of either anti-CD81 (JS-81; 555675, BD Pharmingen, Heidelberg, Germany), anti-CD81 (5A6 #SC28962,SCBT, Heidelberg, Germany), anti-SR-BI (C16-71 gifted from Phillip Meuleman, Ghent, Belgium), or mouse IgG1 κ isotype control (#554721, BD Pharmingen).^31^ After 1 h of incubation at 37 °C, cells were infected with ∼200 focus-forming units (FFU)/well. After further incubation for 3 h, cells were washed once with PBS and fresh media added. At 48 h after infection, cells were fixed with ice-cold methanol and stained for NS5A expression by immunofluorescence staining as described above.

For HCV neutralization, approximately 200 FFU of virus was incubated with a serial dilution of the Fab equimolar mix of AR3C/HC84.1 (gifted from Thomas Krey, Lübeck, Germany), after 1 h at 37 °C, Huh-7.5 cells seeded 24 h before infection in 96-well plates at a density of 2 × 10^4^ were infected with the virus/Fab mix. After further incubation for 3 h, fresh media was added. At 30 h after infection, cells were fixed with ice-cold methanol and stained for NS5A expression by immunofluorescence staining as described above.

### Firefly Luciferase assay

Cells electroporated with HCV subgenomic replicon *in vitro* transcribed RNA were lysed with 30 μl/well of Firefly Luciferase lysis buffer (1% [v/v] Triton X-100, 25 mM diglycine, 15 mM MgSO_4_, 4 mM EGTA, 1% [v/v] DTT in H_2_O) and subsequently frozen at -20 °C. Samples were then thawed at room temperature and 20 μl of lysate were mixed with 72 μl of assay buffer (25 mM diglycine pH 7.8, 15 mM KPO_4_, 15 mM MgSO_4_, 4 mM EGTA pH 8, 1 mM DTT, 2 mM ATP pH 7.8). Forty microliters of substrate solution (0.2 mM D-luciferin, 6 mM diglycine in H_2_O) were automatically injected into each well directly before measurement in the Berthold LB 960 microplate reader (Berthold Technologies, Gütersloh, Germany).

### Quantification of HCV core antigen

HCV viral supernatants were diluted 1:50 in 1% Triton X-100/DMEM and subsequently analyzed using an HCVag chemiluminescent microparticle immunoassay. The Architect HCV Ag reagent kit (#6L47, Abbott Laboratories, IL, USA) and the Architect i2000 immunoassay analyzer were used according to the manufacturer’s instructions.

### Statistical analysis

Statistical analysis was performed using GraphPad Prism V9 (GraphPad Software, San Diego, CA, USA). Statistical significance was tested using one-way Anova using Šídák’s multiple comparison test with a single pooled variance as indicated in the respective figure legend. Significance levels were designated as follows: ∗*p* <0.05, ∗∗*p* <0.01, ∗∗∗*p* <0.001, and ∗∗∗∗*p* <0.0001.

## Results

### Step-wise adaptation of HCV leads to an increased infection in mouse hepatocyte cell line

**Fig. 1:**
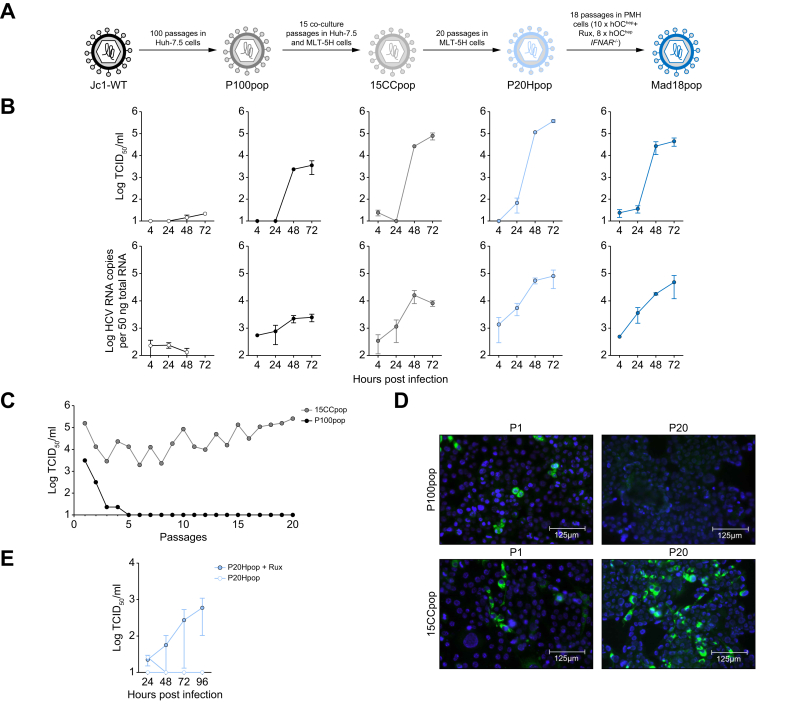
Step-wise adaptation towards mouse hepatocyte tropism. (A) Schematic figure depicting the step-wise adaptation towards mouse hepatocyte tropism. (B) Replication kinetics in MLT−5H cells after inoculation with equal viral genome copies of the given virus populations. Intracellular HCV RNA copies and extracellular infectious virus production were quantified. The data represent the means of three independent experiments ± SEM. (C) MLT-5H cells were infected with 2 TCID_50_/cell with either P100pop or 15CCpop. Every 3–4 days naive cells were infected with the supernatant from the previous passage. Infection was monitored by extracellular infectious virus production. (D) Immunofluorescence staining of HCV NS5A (green) and nuclear staining (DAPI, blue) of MLT-5H cells after one and 20 passages of P100pop or 15CCpop. (E) Extracellular infectious virus production of P20Hpop after infection of plated primary hepatocytes from transgenic mice, hOC^hep^, with or without the treatment of ruxolitinib (Rux, 10 μM). The data represent the means of two individual experiments ± SEM.

Perales *et al.*^32^ developed a cell culture HCV population with a high replication fitness and partial resistance to interferon. Using this virus population (P100pop), we aimed to adapt HCV to efficiently replicate in PMHs (Fig. 1A). First, we tested the replication efficiency of P100pop in a mouse liver tumor cell line (MLT) from Mitochondrial Antiviral Signaling Protein knock out mice (MAVS^-/-^) that over expresses four human HCV entry factors (CD81, SR-B1, CLDN1, and OCLN), human apolipoprotein E (APOE), and the micro RNA, miR-122, (MLT-5H).^19^ P100pop, unlike the parental strain, Jc1-WT, could efficiently infect and release new infectious viral progeny from these MLT-5H cells over 72 h, as was previously shown^26^ (Fig. 1B). However, P100pop was unable to continuously propagate in these cells and was lost after three passages, likely because replication efficiency was insufficient (Fig. 1C). We hypothesized that a larger and more diverse P100pop quasispecies may comprise viral variants with enhanced fitness in murine cells, and that these may ultimately propagate in and further adapt to these cells. Therefore, we attempted to increase P100pop quasispecies diversity by first infecting the highly permissive human Huh-7.5 liver cell line^17^ with P100pop until >99% of the cells were infected. Subsequently, we mixed and plated these cells at a 10:1 ratio with the MLT-5H cells. After 72 h of co-culture, we added antibiotics to selectively eliminate the Huh-7.5 cells and continued passaging the MLT-5H two more times. Then, we collected the supernatant of the surviving MLT-5H cells and used it to inoculate naive Huh-7.5 cells. We repeated this selection procedure, which first creates a vast quasispecies and then pushes it through the bottleneck of poorly permissive murine cells, followed by amplification of remaining viruses in highly permissive human cells, for 15 times. This created a new virus population (15 co-cultures; 15CCpop), which maintained a sustained infection in MLT-5H cells (Fig. 1C). Next, we passaged 15CCpop for 20 times solely in MLT-5H cells generating P20Hpop. Interestingly, 15CCpop, unlike P100pop, maintained high replication and infectious virus release throughout this extensive passaging (Fig. 1C and D). The P20Hpop was then tested for its ability to infect PMHs from transgenic mice expressing human CD81 and OCLN hepatically (hOC^hep^).^26^ The P20Hpop productively infected these cells, however, only when type I interferon signaling was ablated by the JAK 1 inhibitor, Rux (Fig. 1E). We recovered the virus from the supernatant, expanded it on Huh-7.5 cells and subsequently infected naive hOC^hep^ PMH cells with the resulting population in the presence of Rux. Passaging of virus in primary cells in absence of type I interferon signaling was repeated 18 times, first 10 times in hOC^hep^ PMH in presence of Rux and then a further eight times in PMHs of mice that express the human entry factors and which simultaneously have a type 1 interferon receptor knockout (hOC^hep^ IFNAR^-/-^) to create a final mouse-adapted population, Mad18pop. When comparing the infectious virus release and intracellular replication of these five different HCV populations (Jc1-WT, P100pop, 15CCpop, P20Hpop, and Mad18pop) in the MLT-5H cells, we observed strong differences in viral fitness (Fig. 1B). Mad18pop accumulated more than 1,000-fold higher infectious viral progeny and more than 100-fold increased intracellular RNA compared with the parental Jc1-WT population strongly indicating that it had adapted to high replication fitness in these MLT-5H cells.

### HCV adaptation results in enhanced fitness in PMHs

**Fig. 2:**
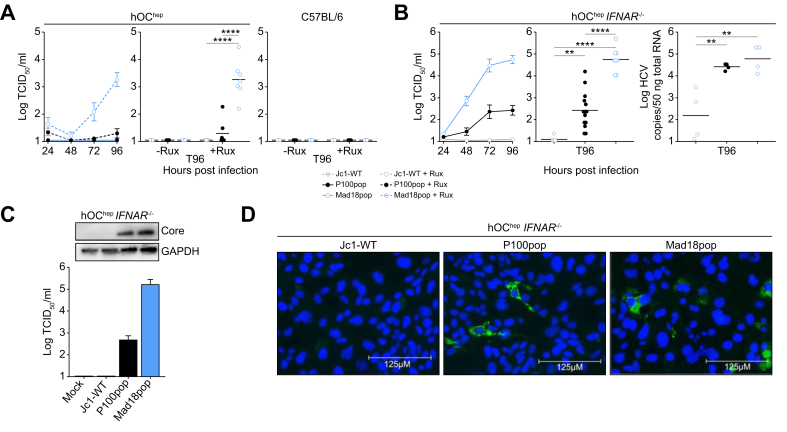
HCV adaptation results in enhanced fitness in primary mouse hepatocyte cell line. (A) Extracellular infectious virus production of Jc1-WT, P100pop, and Mad18pop in plated primary hepatocytes from hOC^hep^ or C57BL/6 mice with or without treatment of 10 μM ruxolitinib (Rux). The data (left) represent the means of three to six individual experiments ± SEM. Comparision of 96 h post infection titers using one-way Anova using Šídák’s multiple comparison test with a single pooled variance, each circle represents an independent experiment. (B) Extracellular infectious virus production of Jc1-WT, P100pop, and Mad18pop in plated primary hepatocytes from hOC^hep^ IFNAR^-/-^ mice. The data (left) represent the means of four to 13 individual experiments ± SEM. Comparison of 96 h post infection titers or intracellular RNA copies/50 ng total RNA using one-way Anova using Šídák’s multiple comparison test with a single pooled variance, each circle represents an independent experiment. Asterisks in (A) and (B) represent statistical significance compared to Jc1-WT (black) or compared P100pop (blue), non-significant comparisons are not shown. (C) Western blot detection of HCV core antigen and GAPDH in plated primary hepatocytes from hOC^hep^ IFNAR^-/-^ mice treated with conditioned media (mock) or infected with Jc1-WT, P100pop, or Mad18pop (2 TCID_50_/cell) for 96 h. The corresponding extracellular infectious virus production is shown below. (D) Immunofluorescence staining of HCV NS5A (green) and nuclear staining (DAPI, blue) on plated primary hepatocytes from hOC^hep^ IFNAR^-/-^ mice infected with Jc1-WT, P100pop, or Mad18pop, (2 TCID_50_/cell) for 96 h. IFNAR, interferon A receptor.

Given that immortalized liver tumor cell lines only partially recapitulate the properties of primary liver cells, we next compared the capacity of Jc1-WT, P100pop, and Mad18pop to infect and propagate in PMHs. Neither of the virus populations studied productively infected hOC^hep^ PMH (Fig. 2A), this is likely because the upregulation of interferon regulated genes as previously observed with Jc1 infection of hOC^hep^ PMH^26^ or P100pop infection of stem cell-derived hepatocyte-like cells^20^ or PHH.^23^ In line with this hypothesis, supplementation of hOC^hep^ PMH infections with Rux or inoculation of hOC^hep^ IFNAR^-/-^ PMH resulted in the accumulation of high numbers of Mad18pop viral progeny up to 5 × 10^4^ TCID_50_/ml (Fig. 2A and B). P100pop also productively infected hOC^hep^ IFNAR^-/-^ PMH, however, peak infectious virus titers were 100-fold lower compared with Mad18pop. In contrast, inoculation of these cells with Jc1-WT barely resulted in production of any detectable infectious virus. Mad18pop infection of hOC^hep^ IFNAR^-/-^ cells was robust and easily detected by qRT-PCR, Western blotting and fluorescence microscopy (Fig. 2B–D). Finally, when we inoculated PMH derived from wild-type C57BL/6 mice in the presence of Rux, we did not detect any infectious viral particles (Fig. 2A). These findings show that Mad18pop, but not Jc1-WT productively infects PMH and that infection depends on ectopic expression of human entry factors and the blunting of innate immune signaling. Furthermore, Mad18pop produces ∼100-fold more infectious particles in hOC^hep^ IFNAR^-/-^ PMH than P100pop, demonstrating that the adaptation has strongly enhanced HCV fitness in PMHs (Fig. 2B). Conclusively, we showed that adaptation of HCV enables the completion of the entire virus replication cycle in PMHs in innate immune deficient cellular environment, including virus spread. To our knowledge, this has not been achieved until now.

### No viral enhancement in human or macaque hepatocytes

**Fig. 3:**
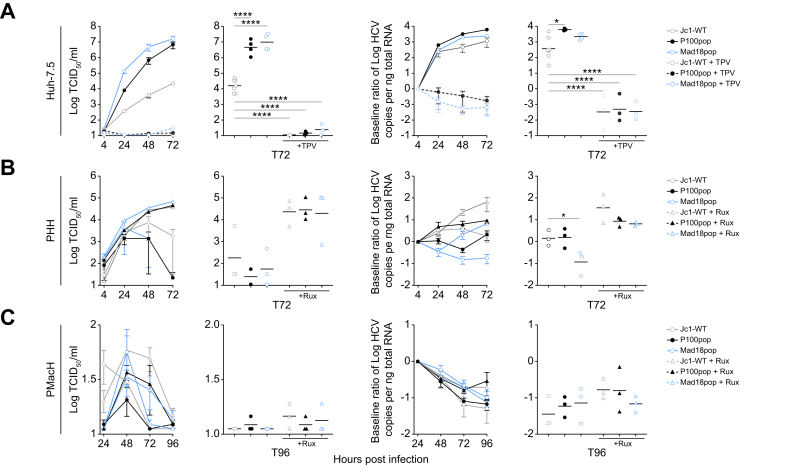
No viral enhancement in human or macaque hepatocytes. Replication kinetics of the viral populations in (A) Huh-7.5 in presence and absence of telaprevir (TPV) (5 μM), (B) PHH in presence and absence of ruxolitinib (Rux) (10 μM), (C) primary macaque hepatocytes in the presence and absence of Rux. Extracellular infectious virus production and intracellular HCV RNA copies were monitored, and data represent the mean of three to six experiments ± SEM. Comparison of 72 h post infection titers or intracellular RNA copies/50 ng total RNA using one-way Anova using Šídák’s multiple comparison test with a single pooled variance, each circle represents an independent experiment. Asterisks represent statistical significance compared to Jc1-WT, non-significant statistics are not shown.

As the adaptation involved a series of passages in both human and mouse cells, we hypothesized that the enhanced replication of Mad18pop in murine liver cells may be because of a general gain of fitness in both human and mouse cells, and potentially liver cells, from other species. To test this, we compared infection kinetics of these HCV populations in Huh-7.5 cells, PHHs, and in PMacHs as these have previously been shown to support HCV infection.^33^ In line with our previous studies, P100pop was much fitter than Jc1-WT in Huh-7.5 cells^20^^,^^32^ (Fig. 3A). Mad18pop accumulated intracellular RNA copy numbers comparable to P100pop, but produced significantly more infectious progeny already at 24 h post infection (h.p.i.) (*p* = 0.014) (Fig. 3A). Supplementation of telaprevir, an HCV NS3-4A protease inhibitor, completely suppressed replication and virus production of all tested populations, confirming that the adaptation did not confer drug resistance (Fig. 3A). Unlike the situation in MLT-5H and in hOC^hep^ PMH, where Mad18pop exhibited a significantly enhanced fitness compared to P100pop (Fig. 1, Fig. 2), in PHHs, and PMacHs, both virus populations had similar fitness, which was comparable to Jc1. These data strongly indicate that the adaptation has selectively enhanced HCV fitness in mouse liver cells but did not enhance HCV fitness in PHHs or PMacHs. Notably, blunting of innate immune signaling by Rux equally increased replication of all three virus populations in PHH cells (Fig. 3B). This suggests that P100pop and Mad18pop evolved improved host factor usage in Huh-7.5, but not in PHHs or PMacHs. In summary, these data show that the adaptation to mouse cells did not further increase tropism to macaque or increase the infectivity and replication in human primary liver cells.

### Sequence analysis of HCV adapted populations

**Fig. 4:**
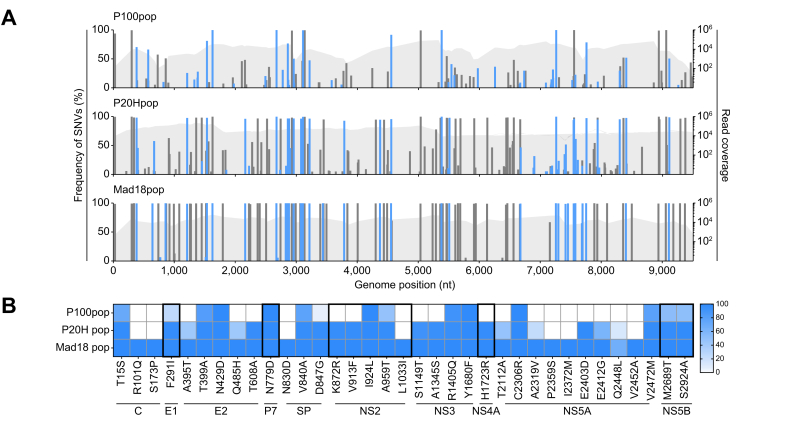
Sequence analysis of adapted HCV populations. (A) Next generation sequencing of the intermediate steps towards Mad18pop (including P100 population, P20H population and Mad18 population), RNA Seq Fastaq files were aligned to the sequence of Jc1-SP (accession number # OQ726017) using CLC Genomics Workbench (Qiagen). Genome coverage is depicted in light grey. Non-synonymous single-nucleotide variants (SNVs) detectable at a population frequency of 5% are highlighted in blue and synonymous SNVs in black. (B) Frequency of non-synonymous mutations in the adapted populations. Protein regions are indicated below. SP, the signal peptide remaining from the initial Gaussia Luciferase of the Jc1 used, also see Frericks *et al.*^23^ and Perales *et al.*^32^

The ability to cross the mouse species barrier is expected to be associated with changes in the viral genome. To pinpoint molecular changes conferring different viral fitness and tropism, we next sequenced the virus populations from each of the adaptation stages. A similar coverage (mean 1 × 10^4^ reads/nucleotide) for each of the populations was generated and low frequency single-nucleotide variants (SNVs) that were <5% were excluded from further analysis. Interestingly, the number of low frequency variants decreased through the adaptation stages and several SNVs became fixed in the consensus sequence (>50% frequency) (Fig. 4). The 12 non-synonymous mutations already present in the consensus sequence of P100pop^23^ remained fixed in Mad18pop suggesting their importance for the general increased fitness in cell culture. Three additional non-synonymous mutations, (E1, F291L and NS2 D847G, and A959T) were detected at low level frequencies (25.1%, 5.2%, and 47.5%, respectively) already in the P100 population but later became dominant in the P20H population. A further seven mutations became fixed in the P20H population. Finally, 13 non-synonymous only became dominant after passaging in the PMH suggesting an important role of these changes for replication in mouse hepatocytes. Interestingly, seven of these 13 mutations reside in domain 2 of the non-structural 5A coding region (Fig. 4). Thus, collectively, Mad18pop carries 35 non-synonymous coding mutations distinguishing it from the Jc1 parental virus.

### Enhanced replication of molecular clones in mouse cells

The sequencing data revealed that 34/35 non-synonymous and 63/66 synonymous mutations in the consensus sequence had frequencies of >90%/>50%, respectively (Fig. 4 and Table S1). This suggested that both types of mutations may cooperate to confer enhanced fitness of Mad18pop. To address this, we created molecular clones, that contained all non-synonymous and synonymous consensus mutations of Mad18pop together (Mad18cl; in total 35 non-synonymous and 66 synonymous changes) or only the non-synonymous (Mad18ns; in total 35 coding mutations). To explore changes in viral fitness between P100cl and Mad18cl, we used the P100 clone (P100cl) previously described.^23^

**Fig 5:**
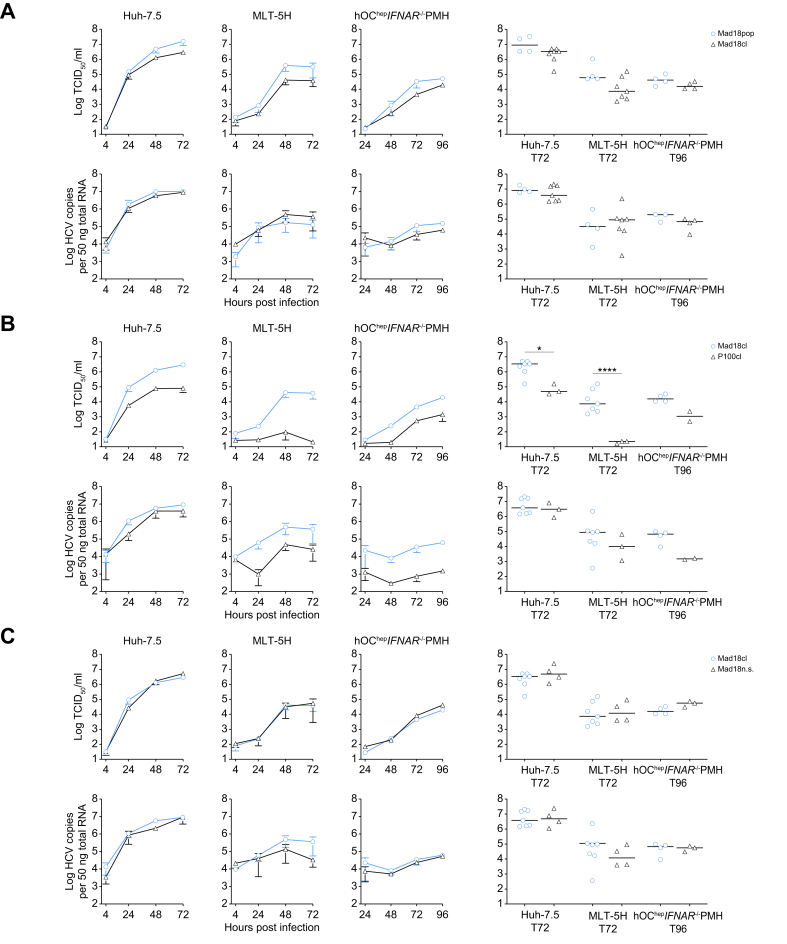
Enhanced replication of molecular clones in mouse cells. Extracellular infectious virus production and intracellular HCV RNA copies were measured in Huh-7.5, MLT-5H, or hOC^hep^ IFNAR^-/-^ PMH and compared between: (A) Mad18pop and Mad18cl; (B) Mad18cl and P100cl; and (C) Mad18cl and Mad18ns. Data represent the means of two to four independent experiments ± SEM. Comparison of 72 or 96 h post infection titers or intracellular RNA copies/50 ng total RNA using one-way Anova using Šídák’s multiple comparison test with a single pooled variance, each circle represents an independent experiment. Asterisks represent statistical significance between each pair, non-significant statistics are not shown. IFNAR, interferon A receptor.

We first compared the infection of the Mad18pop with its molecular clone, derived from the consensus sequence (Mad18cl). A slightly higher replication fitness, but not significant, of the population in both the human (Huh-7.5) and mouse cells (MLT-5H and hOC^hep^ IFNAR^-/-^ PMH) was observed (Fig. 5A).

We next compared the fitness of the molecular clones of Mad18 and P100 and found, similar to the populations (Fig. 1, Fig. 2A), a significantly enhanced fitness of Mad18cl over P100cl particularly in MLT-5H cells. Mad18cl also exhibited increased infectious virus production from Huh-7.5 cells over P100cl enhancing this trend, which was also visible for the populations (Fig. 5B compared with Fig. 3A).

We then compared the Mad18cl, which has both coding and non-coding mutations to the Mad18ns, which only has the coding mutations and found that both clones had almost identical replication finesses in both human and mouse cells (Fig. 5C). Collectively, these results establish a molecular clone of Mad18pop (Mad18cl), which phenocopies the enhanced mouse liver cell tropism of the adapted virus populations. Furthermore, these data show that protein-coding and not non-coding mutations are the main drivers for the increased fitness of HCV in mouse liver cells (Fig. 5C).

Overall, we could successfully show that a molecular clone generated from the consensus sequence of Mad18pop, was fully functional and replicated and infected to a similar level to the population in mouse liver cells with or without the non-coding mutations.

### Enhanced replication in mouse cells without the need of mutations in the glycoproteins

**Fig. 6:**
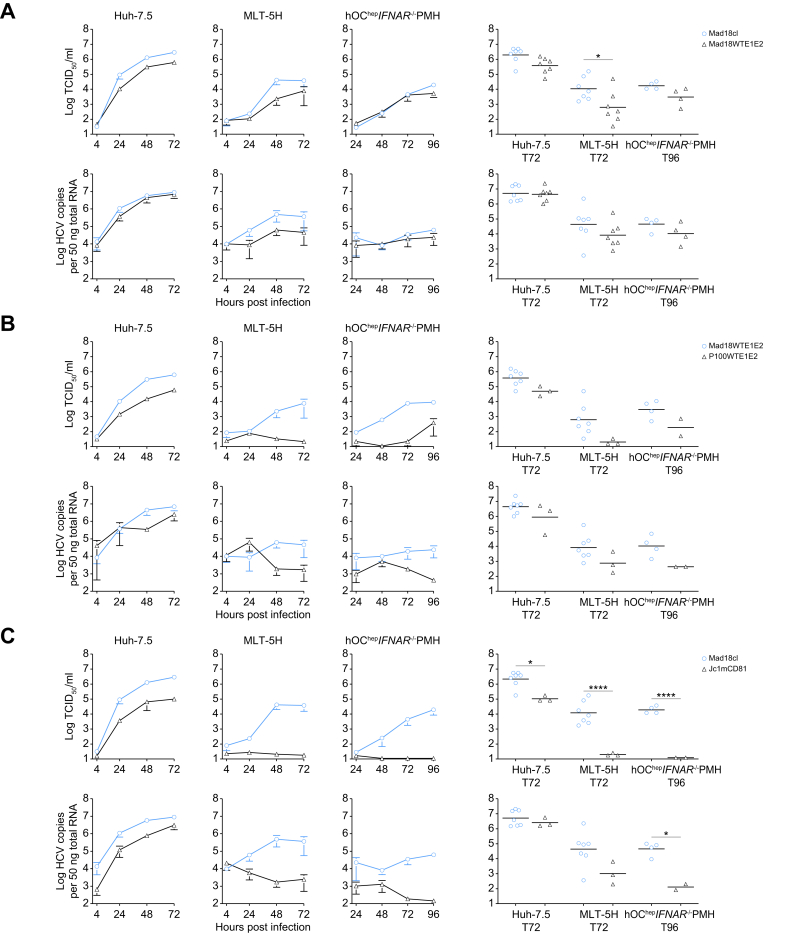
Enhanced replication in mouse cells does not require mutations in the glycoproteins. Extracellular infectious virus production and intracellular HCV RNA copies were measured in Huh-7.5, MLT-5H, or hOC^hep^ IFNAR^-/-^ PMH and compared between: (A) Mad18cl and Mad18WTE1E2; (B) Mad18WTE1E2 and P100WTE1E2; and (C) Mad18cl and Jc1mCD81.^18^ Data represent the means of three to seven individual experiments ± SEM. Comparison of 72 or 96 h post infection titers or intracellular RNA copies/50 ng total RNA one-way Anova using Šídák’s multiple comparison test with a single pooled variance, each circle represents an independent experiment. Asterisks represent statistical significance between each pair, non-significant statistics are not shown. IFNAR, interferon A receptor.

Next, we investigated which enhancements of distinct life cycle stages improved the infection of mouse cells. Entry into the target cell is a critical step for a virus to establish infection. HCV entry is mediated by the interaction of the viral glycoproteins E1 and E2 with the main entry factors CD81 and SR-BI followed by a complex signaling cascade for the induction of clathrin-mediated endocytosis of the viral particle and the use of CLDN1 and OCLN.^7^^,^^34^ To explore whether adaptive mutations in the glycoproteins are critical for enhanced fitness by mediating virus uptake more efficiently, we generated two additional constructs where the sequence of the E1E2 structural proteins was reverted to the wild-type sequence. These clones are referred to as Mad18WTE1E2 and P100WTE1E2, respectively. We compared the replication kinetics of the Mad18cl with the new Mad18WTE1E2 clone. In all three cell culture systems there was a small reduction in viral infectivity of the Mad18WTE1E2 clone, which was only significant in the MLT-5H cells (Fig. 6A). However, unlike the parental clone (Jc1-WT) (Fig. 2A and B), infection was still possible in the primary mouse hOC^hep^ IFNAR^-/-^ hepatocytes (Fig. 6A), suggesting that E1E2-dependent changes of cell entry are not the main drivers of adaptation towards mouse liver cells. This could also be confirmed when we compared the P100WTE1E2 clone with the Mad18WTE1E2 and like the clones (Mad18cl and P100cl), Mad18WTE1E2 was considerably fitter than the P100WTE1E2 in both human and mouse cells, suggesting again, mutations outside of the E1E2 are responsible for the mouse adaptation (Fig. 6B). Adaptation of HCV glycoproteins to murine CD81 (mCD81) has previously been shown to facilitate usage of other species’ CD81 for entry of the adapted HCV.^18^ Therefore, we also compared the Jc1 mouse CD81 adapted clone, Jc1mCD81, which includes four mutations in E2 and p7 (L216F, V388G, M405T, and N767D) with the Mad18cl. We could show a much more robust replication and infectivity of the Mad18cl in both MLT-5H cells and the primary mouse hOC^hep^ IFNAR^-/-^ hepatocytes indicating adaptation to mCD81 is not sufficient for an improved tropism towards mouse (Fig. 6C).

### Adaptation increases neutralization sensitivity and alters receptor usage

**Fig. 7:**
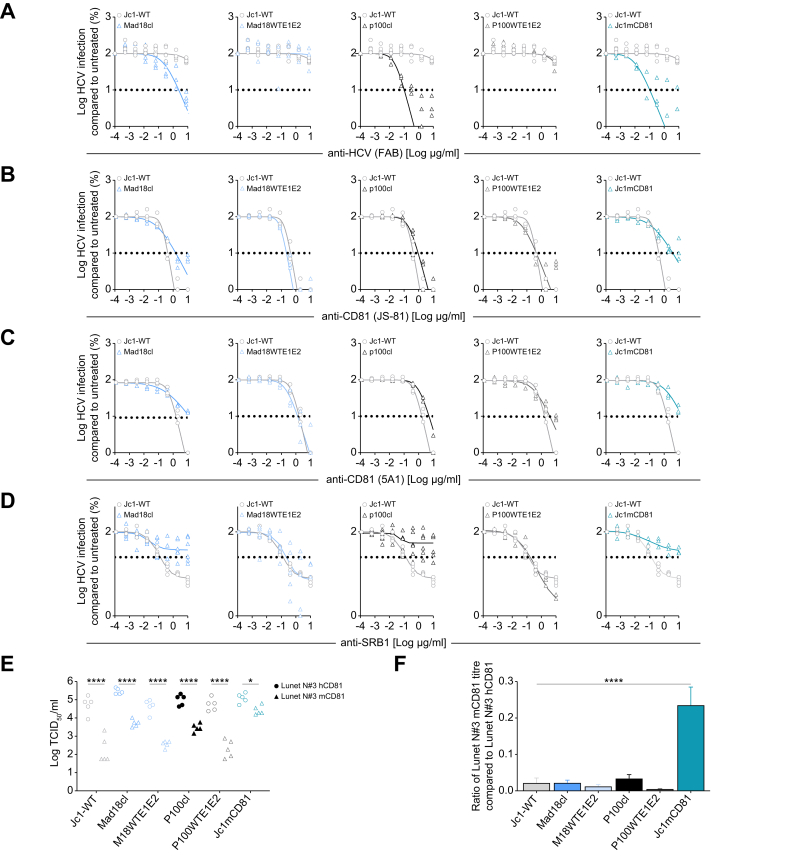
Adaptation increases neutralization sensitivity and alters receptor usage. (A) Percentage infectivity of molecular clones compared to Jc1-WT after neutralization of HCV clones with increasing concentrations of anti-HCV E1E2 Fab mix (AR3C/HC84.1). (B) Percentage infectivity of molecular clones compared to Jc1-WT after antibody binding of Huh-7.5 cells with increasing concentrations of anti-CD81 (JS-81) (B), anti-CD81 (5A1) (C), or anti-SR-B1 (D). Data represent the means of three to five individual experiments. Horizontal dashed line represents the IC_90_ (A–C) or IC_50_ (D). (E) Virus titration of molecular clones on Lunet N#3 cells exogenously expressing hCD81 or mCD81. Each circle/triangle represents an individual experiment, asterisks represent statistical significance compared to Lunet N#3 hCD81 cells using one-way Anova using Šídák’s multiple comparison test with a single pooled variance. (F) Data from panel (E), infectious titers of Lunet N#3 mCD81 cells were normalized to titers on Lunet N#3 hDC81 cells. Data represent the means ± SEM, asterisks represent statistical significance compared with Jc1-WT one-way Anova using Šídák’s multiple comparison test with a single pooled variance.

We have previously shown that HCV adaptation to murine CD81 resulted in glycoprotein changes, which enhance access to the viral CD81 binding site and increase neutralization by E1E2 specific antibodies.^18^ Therefore, we next compared the susceptibility of the different clone-derived HCV particles to HCV neutralizing antibodies. We used a mix of antigen-binding fragments (Fabs) of the HCV neutralizing antibodies AR3C and HC84.1 known to be potent HCV neutralizers.^35^^,^^36^ Mad18cl, Jc1mCD81, and P100cl were much more sensitive to neutralization compared with Jc1-WT (Fig. 7A and Table S2), as shown by IC_50_ (μg/ml) values of 0.372, 0.024, and 0.016 μg/ml, respectively, compared with >10 μg/ml for Jc1. However, when the E1E2 sequence of either P100 or Mad18 was reverted to the parental Jc1 sequence, neutralization susceptibility was reverted to levels similar to Jc1-WT (>10 μg/ml) without a great loss in fitness in mouse cells (Fig. 6A and B). Taken together, these results show that the mutations within the glycoproteins of the Mad18 and P100 clone facilitate neutralization.

Using CD81 antibody competition assays we could further show that Jc1-WT and P100cl had similar dependency on CD81 using two different antibodies (JS-81 and 5A1) while the Jc1mCD81 and Mad18cl were more resistant to CD81-binding antibodies (Fig. 7B and C and Table S2). Resistance to SR-B1-targeting antibodies was clearly enhanced for P100, Mad18, and Jc1-mCD81 clone-derived particles compared with Jc1-WT (Fig. 7D and Table S2). Similar to the viral neutralization by the HCV neutralizing Fab mix, the sensitivity was restored when the glycoprotein mutations were reverted to the Jc1-WT sequence, suggesting the mutations in the envelope proteins modulate the viral SR-B1 usage.

Even though our previous experiments indicated that mutations outside of the envelope proteins are important for the increased fitness in mouse cells, Mad18 may use murine CD81 (mCD81) more efficiently than Jc1-WT, mediated by the mutations in the viral glycoproteins. To test this hypothesis, we used a Lunet subclone (Lunet N#3) with no detectable endogenous CD81 expression and either hCD81 or mCD81 exogenously expressed^18^ and titrated the different clone-derived viruses on these cell lines. A comparison of the virus titers on these cells presenting either hCD81 or mCD81 provides an estimate of the species-specific CD81 receptor usage. As previously reported, Jc1mCD81 infectiousness was only ∼five-fold different between Lunet N#3 mCD81 and hCD81 cells. In contrast, titers differed ∼100-fold for Jc1-WT confirming species-specific CD81 usage of Jc1 and mouse CD81 adaptation of Jc1mCD81 (Fig. 7E and F).^18^ In summary, we observed increased neutralization susceptibility in combination with altered CD81 and SR-BI dependency for both the P100 and Mad18 clones. Taken together with our previous findings this suggests only a minor contribution of the adaptive mutations in the glycoproteins. However, they are not the main driver of mouse adaptation (Fig. 6A), but rather enhance cell culture infection.

### Increased specific infectivity in mouse cells

**Fig. 8:**
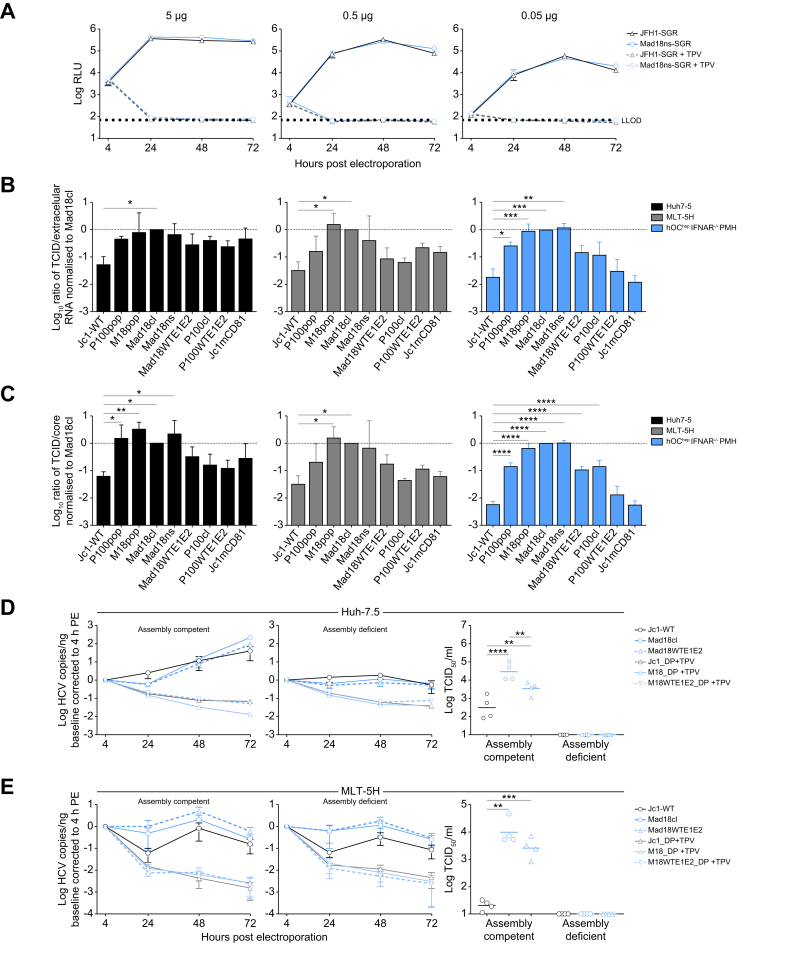
Increased specific infectivity in mouse cells. (A) Measurement of luciferase activity (relative light units, RLU) in lysates from MLT-5H cells electroporated with subgenomic replicons, JFH1 and Mad18ns ± TPV (1 μM). The data represent the mean ±SEM of four individual experiments. (B) Bar graph representing the ratio between extracellular HCV RNA and infectious virus particle release of the different viral populations or clones ± SEM of two to seven individual experiments, 72 h post infection of either Huh-7.5 cells or MLT-5H cells and 96 h post infection of HOC ^hep^ IFNAR^-/-^ PMH. (C) Specific infectivity of the populations and molecular clones calculated by dividing the TCID_50_/well by the concentration of core (fmol)/well and then normalized to Mad18cl ± SEM of two to seven individual experiments, from Huh-7.5 cells (left), MLT-5H cells (middle), or HOC^hep^ IFNAR^-/-^ PMH (right). Stars represent statistical significance compared to Jc1-WT using one-way Anova using Šídák’s multiple comparison test with a single pooled variance. Replication kinetics measured by HCV RNA copies normalized to the 4 h time point post electroporation in either: (D) Huh-7.5 cells (top) or (E) MLT-5H (bottom) using assembly-competent RNA transcripts of Jc1-WT, Mad18 clone, or Mad18WTE1E2 (left panel) or clones harboring a double proline mutation in the core protein rendering the virus assembly deficient (middle panel) and infectious virus release 72 h post electroporation (right panel) with or without 1 μM TPV. To facilitate quantification of virus spread, limiting quantities of RNA were transfected (*i.e.* 0.05 μg and 0.5 μg for Huh-7.5 and MLT5H cells, respectively). The data represent the mean ± SEM of four individual experiments. IFNAR, interferon A receptor; LLOD, lower limit of detection; TPV, telaprevir.

In the previously described experiments, the difference in the production of infectious viruses between the Mad18pop and parental Jc1 clearly exceeded the difference between these viruses regarding the accumulation of intracellular viral RNA (Fig. 2, Fig. 3A).This does not only suggest an effect of the mutations on replication, but also on other steps of the viral life cycle. To test this more directly, we created a Mad18ns-based NS3–5B replicon (Mad18ns-SGR) and compared its RNA replication capacity with one of the parental JFH-1 SGR upon transfection of MLT-5H cells (Fig. 8A). Replication of both SGRs was identical across several different doses of transfected input RNA. This finding indicated that – at least in the context of replicons – the coding mutations in the Mad18ns-NS3-5B region did not affect the efficiency of RNA translation, RNA replication nor RNA stability. Next, we analyzed and compared the intra- and extracellular RNA amounts as well as extracellular levels of core protein – a measure for newly secreted virus particles – 72 h after inoculation of Huh-7.5 and MLT-5H cells and 96 h after infection of PMH hOC^hep^ IFNAR^-/-^ PMH with equal numbers of infectious HCV particles (Fig. S1). Interestingly, both, intra- and extracellular HCV RNA levels were similar across all the clones and populations for infection of Huh-7.5 cells. This suggested that each virus had replicated to saturating levels in these highly permissive human cell line at this time point. Similarly, also total accumulation of HCV RNA was comparable (Fig. S1). Contrary to this, particle release (core) and, to a larger extent, infectious particle release (TCID_50_) was increased for the Mad18pop (>10-fold more core release, 100-fold more infectious particle release), Mad18cl, Mad18ns, Mad18WTE1E2, and P100pop compared with Jc1-WT (Fig. S1). This suggests that more particles are released and that these are more infectious. To quantify this more precisely, we compared the ratio of infectious virus particles per extracellular HCV RNA and per extracellular core protein. This ratio quantifies the ‘specific infectivity’ associated with HCV RNA genome equivalents or core proteins, respectively. Using either RNA or core to estimate particle numbers, we found a significant increase for the mouse-adapted population and clones compared with Jc1-WT, suggesting that virus particles released from Mad18 infected cells are more infectious than that released from Jc1-WT infected cells (Fig. 8B and C). This observation of increased specific infectivity was reproduced in MLT-5H cells and particularly prominent in PMH. Interestingly, the specific infectivity was also higher for Mad18WTE1E2. Although the effect size was smaller and not statistically significant, this trend suggests a contribution of mutations outside of the glycoproteins for release of more infectious particles.

Finally, we used a previously described viral mutant in which two proline residues within the HCV core protein are replaced by alanine (P138A combined with P143A). Mutant viruses carrying this double proline mutation (Core DP) were shown to replicate after transfection but were unable to produce infectious progeny.^37^^,^^38^ This defect is because these mutations prevent the core protein from being transported to lipid droplets.^37^ Under conditions where large amounts of transcripts are transfected (5 μg as in Matthaei *et al.*^38^) and most of the cell takes up and replicates the viral RNA, the spread of viral progeny has little effect on the total amount of viral proteins (*e.g.* core or NS5A) and therefore the mutant genomes accumulate similar amounts of viral proteins, showing that these genomes are replication competent.^37^^,^^38^ Here, we introduced these mutations into the core region of the three full-length constructs (Jc1-WT, Mad18cl, and Mad18WTE1E2) to assess differences in the replication kinetics of the full-length genomes in the presence or absence of indirect effects caused by infectious virus production and secondary rounds of infection. To quantify effects on RNA replication, we monitored the accumulation of viral RNA following transfection in the presence or absence of TPV. In addition, to improve quantification of potential viral spread, we transfected very small amounts of viral RNA (0.05 μg RNA for Huh-7.5 and 0.5μg for MLT-5H cells, *i.e.* 10–100 × less than in Matthaei *et al.*^38^). These viral transcripts with or without the double proline mutations were electroporated into Huh-7.5 or MLT-5H cells and RNA accumulation and infectious virus particle production were quantified (Fig. 8D and E). All three assembly-competent clones exhibited similar kinetics of intracellular RNA accumulation in the transfected Huh-7.5 cells, exceeding the RNA abundance in the TPV-treated controls by approximately 100-fold at 72 h (Fig. 8D). Notably, the assembly-deficient clones also replicated but accumulated only five-to 10-fold more RNA compared with the TPV controls, suggesting that under conditions of low RNA transfection, secondary rounds of infection had strongly contributed to the accumulation of intracellular RNA in Huh-7.5 cells.

As expected, the assembly-competent Mad18 clones had released significantly more infectious particles compared to the Jc1-transfected cells (Fig. 8D, right panel). In MLT-5H cells, there was a reproducible, albeit not significant trend that both the assembly-competent and the assembly-deficient Mad18 clones accumulate more intracellular RNA over time compared with Jc1-WT (Fig. 8E). This difference was almost identical between the assembly-competent and the assembly-deficient viral clones, indicating that this was a result of improved RNA replication of the Mad18 clones compared with Jc1-WT in the MLT-5H cells. Interestingly, there was almost no difference in RNA accumulation between assembly-competent and assembly-deficient clones in the MLT-5H cells, suggesting that in this short time-course secondary rounds of infection played no major role. Finally, also in the transfected MLT-5H cells the assembly-competent Mad18 clones produce significantly more infectious progeny compared with the Jc1-WT parent construct (Fig. 8E, right panel).

These results indicate that adaptive mutations within Mad18cl enhance the numbers and specific infectivity of released HCV particles from both Huh-7.5 and from MLT-5H cells (Fig. 8B–E and S1). Interestingly, this was also true for Mad18WTE1E2, albeit to a lesser extent, indicating that these effects are caused, at least in part, by mutations outside of the E1E2 proteins. In addition to this, the adaptive mutations also facilitate RNA replication of transfected, assembly-defective full-length genomes selectively in the mouse liver cells (Fig. 8E). This effect combined with production of greater numbers of particles and their increased specific infectivity promotes viral fitness in mouse liver cells (Fig. 8E). Taken together, these data suggest that the adaptive mutations outside of the E1E2 region increase RNA genome replication of full-length but not SGR HCV RNAs in mouse liver cells but not in the Huh-7.5 cell line. Furthermore, mutations outside of the E1E2 region enhance release of viral progeny and their infectiousness in both mouse and human liver cells. These latter effects are further enhanced by E1E2 mutations. In combination, these effects allow robust infection and HCV propagation of Mad18cl and Mad18WTE1E2 in the context of innate immune incompetent primary mouse liver cells.

## Discussion

In this study, we developed a mouse-adapted HCV, efficiently infecting PMHs deficient in innate immune signaling. This was possible because of step-wise adaptation taking advantage of, firstly, an already cell culture-adapted HCV population (P100pop);^32^^,^^39^ secondly, the use of a highly HCV-permissive human liver cell line (Huh-7.5); thirdly, infection of co-cultures of Huh-7.5 and a murine liver cell line with human HCV cell entry factors and blunted antiviral immunity as a result of ablation of the MAVS gene;^19^ and finally the use of PMHs.

During this virus adaptation, synonymous and non-synonymous mutations have gradually accumulated (Fig. 4). Using different Mad18 clones, we were able to show that the non-synonymous mutations, but not the synonymous mutations, are responsible for the increased viral fitness in mouse liver cells (Fig. 5). This shows that functional changes in viral proteins mediate HCV mouse adaptation. Several synonymous mutations also became dominant in the viral population, possibly as a result of population bottlenecks that occurred during the adaptation process. During these bottlenecks, where phenotypically essential non-synonymous mutations allow viral spread of a particular variant, co-occurring synonymous mutations in the same variant are fixed in parallel. We therefore suggest that many neutral effect mutations, also known as genetic hitchhikers, are selected along the way.^16^^,^^40^ Other authors have shown that synonymous mutations can also influence the replication fitness of HCV. Such mutations may be located in the 5′ untranslated region (5′ UTR) of the HCV genome.^41^^,^^42^ However, these mutations promote HCV fitness only under circumstances of low endogenous micro RNA-122 (miRNA-122) expression by changing the riboswitch properties of the 5′ UTR.^41^ In contrast, engineered changes in the abundance of CpG or UpA dinucleotides have been observed to affect RNA replication and antiviral immunity, thus modulating replication of echoviruses and HCV.[43], [44], [45] However, to our knowledge such profound changes of dinucleotides have not been observed in natural viral selection processes and seem not to play a role in Mad18 adaptation.

Rather, our data show that the non-synonymous exchanges in the envelope protein genes (E1 and E2) contribute to HCV fitness in human and mouse liver cells, but that they are not essential for the cross-species transmission of HCV to mouse liver cells (Fig. 6, Fig. 8). These envelope protein mutations occurred despite the use of murine cells overexpressing the human HCV cell entry factors. Similar to previously described envelope protein mutations after long-term cultivation of HCV in human cell lines, these mutations increase viral fitness and concomitantly affect the utilization of the SR-B1 receptor as well as CD81 and neutralization by envelope protein-specific antibodies (Fig. 7).^18^^,^^46^^,^^47^ Presumably, these mutations are selected because antibody immune pressure is absent and because they facilitate cell entry of HCV. This is probably made possible by an opened conformation of the E1E2 envelope proteins, which exposes the CD81 binding site and facilitates the utilization of this receptor.^18^ Similar to an HCV variant selected by direct adaptation to usage of murine CD81, Mad18 is also more easily neutralized by anti-HCV antibodies that bind conserved epitopes partially occluded by the viral hypervariable region 1 (HVR1) in wild-type HCV. Likewise, for both viruses, a higher anti-CD81 antibody concentration is needed to block cell entry. However, in contrast to mutations generated during targeted adaptation to the murine CD81 ortholog, the envelope protein mutations of Mad18 did not increase the utilization of mouse CD81 (Fig. 7F). Conversely, the HCV variant with these mouse CD81-adaptive mutations (Jc1mCD81)^18^ is highly capable of using mouse CD81, but unable to replicate in mouse liver cells (see Fig. 6, Fig. 7 and Bitzegeio *et al.*^18^ and von Schaewen *et al.*^48^). These findings suggest that additional non-synonymous mutations outside of the envelope proteins play an essential role in mouse adaptation. The data with the P100WT1E2 and Mad18WTE1E2 clones with wild-type envelope proteins support this as the Mad18WTE1E2 clone is substantially superior to Jc1 virus and also to the P100WTE1E2 clone in RNA replication and virus production in murine liver cells (Fig. 6 and S1). The ability to insert non-adapted envelope proteins into the MAD18 clone without losing the adaptation to mouse cells could be used in the future to generate viruses with different envelope proteins, thus extending the range of applications of the system, for example in vaccine research. The phenotypic comparisons of the different full-length clones in human and murine cells show that the Mad18 mutations slightly enhance RNA replication but primarily increase the production and release as well as the infectivity of released virus particles in human and murine liver cells (Fig. 3, Fig. 8, and S1). Notably, several mutations in NS2, NS3, core, NS4A, and especially 5A differentiate the Mad18 population from the P100 population by appearing only after passage in murine cells (Fig. 4). The relevance of mutations of the non-envelope proteins was shown by various studies indicating that, in addition to core, viral non-structural proteins such as NS2 and also the NS3/4A protease and especially NS5A influence the production of infectious viruses.^49^ In particular, the accumulation of mutations in domain 2 of NS5A could be of relevance as this region interacts with several host proteins including cyclophilin A (CypA)^50^ and casein kinase II,^51^ as well as NS5B and RNA via an RNA binding motif.^52^ Of note, cyclophilin A has an essential role in HCV replication^53^ and mouse cyclophilin A has previously been shown to not support a robust HCV infection unless two amino acid changes are inserted to make a humanized version of CypA.^54^ One could hypothesize that Mad18 may have adapted to the mouse ortholog of CypA or at least more openly binds/interacts with both human and mouse CypA. Interestingly, one mutation is located in the cyclophillin A-binding domain^50^ at position A2419V, changing the motif from PAWA to PVWA.

Numerous studies have shown adaptation of diverse HCV variants of different genotypes to replication in human cell lines and thereby opened up new avenues for dissecting the HCV life cycle *in vitro*.^55^ However, to our knowledge, the Mad18 population and clones described here, are the first HCV variants that are adapted to mouse liver cells and can efficiently replicate in PMHs while still dependent on critical human host factors, occludin and CD81. This efficiency is documented by the detection of HCV proteins by standard methods and the accumulation of up to about 1E5 TCID_50_/ml, more than 1,000-fold more than Jc1 in the same period (Fig. 2). Until today, only a few studies have observed weak HCV replication in genetically engineered murine cell lines.^56^ The furthest progress towards a mouse model for HCV has been made by Dorner, who showed that low HCV Jc1 viremia can be achieved in receptor transgenic animals with a blunted interferon system.^9^ Because of the significantly increased efficiency of Mad18, completely new possibilities and opportunities arise to develop an HCV animal model, which could significantly boost the preclinical validation of HCV vaccine candidates and also the analysis of the immunological control mechanisms of HCV as well as HCV pathogenesis. In the context of HCV vaccine development, it is relevant that the changes in envelope proteins are not crucial for adaptation to infection of primary mouse liver cells. This opens up the possibility of using adapted HCV variants with wild type, and thus completely authentic envelope proteins as challenge viruses for the qualification of vaccines *in vivo*. Others and we have described various chimeric HCV constructs^21^^,^[57], [58], [59] suggesting that eventually various Mad18-based envelope chimeras could be generated to create genetically diverse HCV challenge viruses. Finally, the Mad18 population and the Mad18 clones provide new opportunities to study the basic principles of HCV species tropism.

It is also important to consider the safety aspects of Mad18 HCV. In our adaptation strategy, we specifically used cells with an inactivated interferon (IFN) system: on the one hand to facilitate adaptation, on the other hand to not enforce a targeted viral evasion of cellular antiviral control mechanisms. As a result of this strategy, Mad18 remains highly sensitive to the effect of interferons. Moreover, Mad18 is dependent on the overexpression of human HCV entry factors and is unable to infect primary hepatocytes of wild-type mice even when Rux suppresses the IFN response (Fig. 2). In addition, our results show that Mad18 is inhibited by TPV, a classical anti-HCV drug, and does not exhibit altered fitness in PHHs. Mad18 is also inhibited by the interferon system in PHHs and PMacHs. These aspects document essential safety features of Mad18 and indicate that neither virulence, nor immune evasion are altered in human cells. At the same time, these *in vitro* findings indicate that further adaptations *in vivo* are probably required to eventually develop a mouse-adapted HCV infecting a fully immunocompetent animal. Further genetic humanization of the mouse model may also be needed and is currently under investigation. Nevertheless, this work demonstrates the feasibility in principle of HCV adaptation to replication in and infection of non-human cells. This is made possible by a manageable number of non-synonymous mutations and opens up new ways to elucidate the principles of HCV species tropism and to develop important animal models for HCV research in the long term.

## Abbreviations

APOE, human apolipoprotein E; CLDN1, claudin-1; Fabs, antigen-binding fragments; FFU, focus-forming unit; IFN, interferon; IFNAR, interferon A receptor; miRNA-122, micro RNA-122; MSM, men who have sex with men; OCLN, occludin; PHH, primary human hepatocyte; PMacH, primary macaque hepatocyte; PMH, primary mouse hepatocyte; PWID, people who inject drugs; Rux, ruxolititnib; SNVs, single-nucleotide variants; SR-B1, scavenger receptor class B type 1; TPV, telaprevir.

## Financial support

Deutsche Forschungsgemeinschaft (DFG, German Research Foundation) under the Germany’s Excellence Strategy – EXC 2155 “RESIST” – project number 390874280 (to TP). National Institute for Allergy and Infectious Disease (R01AI107301) (to TP, JS). German Center of Infection Research in the DELPHI project (TTU 0.5.910) (to TP). European Research Council ERC-2011-StG_281473-(VIRAFRONT) (to TP). Hannover Biomedical Research School (HBRS) and the Center for Infection Biology (ZIB) (to MW, NF). National Research Platform for Zoonoses grant VIRASCREEN (to RJPB).

## Authors’ contributions

Conceptualization: JAS, MW, DW, TP. Methodology: JAS, MW, RJPB, DW, TP. Validation: JAS, MW, KR, DW, TP. Formal analysis: JAS, MW, QY, NF, RJPB, CM. Investigation: JAS, MW, CM, NG, SB, KR, GM. Data curation: JAS, MW, RJPB. Writing (original draft): JAS, TP. Writing (review and editing): JAS, MW, QY, NF, RJPB, CM, NG, SM, KR, GM, FV, DW, TP. Visualization: JAS, MW. Resources: FV, TP. Supervision: JAS, RJPB, DW, TP. Project administration: JAS, DW, TP. Funding acquisition: RJPB, TP.

## Data availability statement

The authors confirm that the data supporting the findings of this study are available within the article and its supplementary materials.

## Conflicts of interest

The authors declare no conflicts of interest that pertain to this work.

Please refer to the accompanying ICMJE disclosure forms for further details.
