# Supplementary material for: Adapted hepatitis C virus clone infects innate immunity-deficient mouse hepatocytes with minimal human HCV entry factors
Source: JHEP Rep. 2025 Jan 18;7(5):101328. doi: 10.1016/j.jhepr.2025.101328 (PMC11999267; doi:10.1016/j.jhepr.2025.101328)
Supplement: Multimedia component 1 [file mmc1.pdf]

Adapted hepatitis C virus clone infects innate immunity-deficient mouse hepatocytes with minimal human HCV entry factors

Julie Ann Sheldon, Melina Winkler, Qinggong Yuan, Nicola Frericks, Richard John Phillip Brown, Csaba Miskey, Natascha Gödecke, Sara Behme, Katharina Rox, Giorgia Mysegades, Florian Vondran, Dagmar Wirth, Thomas Pietschmann

Table of contents

Fig. S1……………………………………………………………………………………...2

Table S1…………………………………………………………………………………4

Table S2………………………………………………………………………………..19

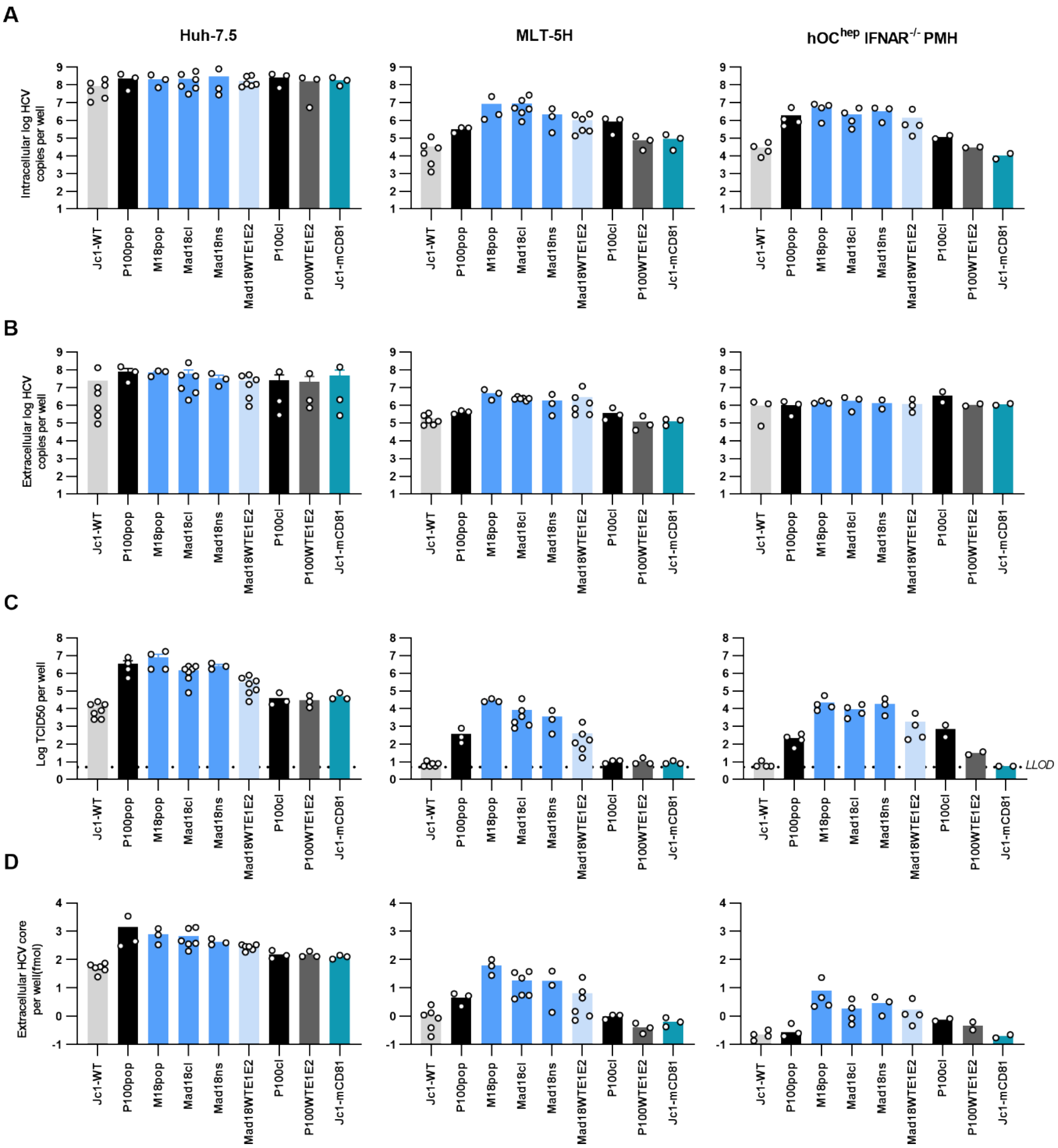

Fig. S1: Measurements of extracellular particle release (core and infectious virus), intra- and extracellular RNA copies numbers of the adapted populations and clones. A. Intracellular HCV RNA quantification, 72h (Huh-7.5 and MLT-5H cells) or 96h (hOC^{hep} IFNAR$^{-/-}$ PMH) post infection. **B.** Extracellular HCV RNA quantification, 72 h (Huh7.5 and MLT-5H cells) or 96h (hOC^{hep} IFNAR$^{-/-}$ PMH) post infection. **C.** Extracellular HCV infectious virus release measured by limiting dilution assay, 72h (Huh-7.5 and MLT-5H cells) or 96h (hOC^{hep} IFNAR$^{-/-}$ PMH) post

infection. **D.** Extracellular HCV core particle quantification, 72 h (Huh-7.5 and MLT-5H cells) or 96h (hOC^{hep} IFNAR$^{-/-}$ PMH) post infection. The data represent the means of 2- 7 biological replicates ±SEM. Circles/triangles represent the individual biological replicates.

Table S1. Table of nucleotide changes of the adapted viral populations. Next generation sequencing of the intermediate steps towards Mad18pop (including P100 population, p20H population and Mad18 population), RNA Seq Fastaq files were aligned to the sequence of Jc1-SP (accession number # OQ726017) using CLC Genomics Workbench (Qiagen). Each table lists the nucleotide position and change (allele), and amino acid position/change according to the position of Jc1-SP (# OQ726017) and H77 (accession number # NC_004102.1).

p100pop ref to H77	ref to Jc1	ref to Jc1-SP	Reference	Allele	Frequency	Coding	Coding region	aa(H77)	aa (Jc1sp)
**29**	28	28	G	A	**94.0**	no	5UTR		
**302**	301	301	T	C	**99.5**	no	5UTR		
**384**	383	383	A	T	**70.5**	yes	Core	15	T15S
**392**	391	391	T	C	**13.2**	no	Core		
**541**	540	540	A	G	**16.3**	no	Core		
**573**	572	572	A	G	**66.2**	Yes	Core	78	K78E
**575**	574	574	A	G	**12.5**	no	Core		
**683**	682	682	T	G	**5.6**	no	Core		
**728**	727	727	C	T	**57.4**	no	Core		
**823**	822	822	G	A	**10.4**	Yes	Core	161	G161E
**860**	859	859	C	T	**51.1**	no	Core		
**902**	901	901	C	T	**9.2**	no	Core		
**1212**	1211	1211	T	C	**21.8**	**Yes**	**E1**	291	F291I
**1212**	1211	1211	T	G	**25.9**	**Yes**	**E1**	291	F291V
**1329**	1328	1328	G	A	**14.6**	Yes	E1	330	A330T
**1380**	1379	1379	A	C	**17.6**	Yes	E1	347	I347L
**1386**	1385	1385	A	G	**28.1**	Yes	E1	349	S349G

**1500**	1499	1535	A	G	**81.2**	Yes	E2	387	T399A
**1535**	1534	1570	C	T	**10.0**	no	E2		
**1590**	1589	1625	A	G	**99.9**	Yes	E2	417	N429D
**1769**	1768	1804	T	C	**7.5**	no	E2		
**1919**	1924	1960	T	G	**18.3**	no	E2		
**1935**	1940	1976	A	G	**7.4**	Yes	E2	532	N546S
**2063**	2071	2107	C	T	**46.2**	no	E2		
**2111**	2122	2158	T	C	**48.5**	no	E2		
**2132**	2143	2179	C	T	**6.0**	no	E2		
**2427**	2438	2474	A	G	**5.3**	no	E2		
**2445**	2456	2492	A	T	**20.5**	Yes	E2	702	M718L
**2462**	2473	2509	T	C	**13.4**	no	E2		
**2505**	2516	2552	T	C	**36.2**	no	E2		
**2517**	2528	2564	T	G	**18.5**	no	E2		
**2628**	2639	2675	A	G	**99.8**	Yes	P7	763	N779D
**2730**	2741	2777	A	G	**31.2**	Yes	P7	797	S813G
		2859	T	C	**76.8**	Yes	Sig Pep		V840A
		2868	A	G	**14.0**	Yes	Sig Pep		L843S
		2880	A	G	**5.2**	Yes	Sig Pep		D847G
**2819**	2830	2926	C	T	**99.1**	no	NS2		
**2850**	2861	2957	A	G	**50.4**	Yes	NS2	837	T873A
**2857**	2868	2964	T	C	**9.0**	Yes	NS2	839	L875P
**2885**	2896	2992	T	C	**24.1**	no	NS2		
**2990**	3001	3097	T	C	**46.7**	no	NS2		
**3003**	3014	3110	A	C	**99.6**	Yes	NS2	888	I924L
**3029**	3040	3136	T	G	**99.6**	no	NS2		

**3108**	3119	3215	G	A	**47.5**	Yes	NS2	923	A959T
**3110**	3121	3217	T	A	**8.8**	Yes	NS2	924	L960M
**3121**	3132	3228	A	G	**9.2**	Yes	NS2	927	Q963R
**3260**	3271	3367	C	T	**9.8**	no	NS2		
**3269**	3280	3376	C	T	**9.7**	no	NS2		
**3472**	3483	3579	T	C	**13.4**	Yes	NS3	1044	V1080A
**3539**	3550	3646	T	G	**16.4**	no	NS3		
**3631**	3642	3738	T	C	**5.8**	Yes	NS3	1097	V1133A
**3663**	3674	3770	T	C	**29.6**	no	NS3		
**3713**	3724	3820	G	A	**42.8**	no	NS3		
**3791**	3802	3898	A	G	**21.4**	no	NS3		
**4136**	4147	4243	G	A	**34.3**	no	NS3		
**4391**	4402	4498	A	G	**28.4**	no	NS3		
**4447**	4458	4554	G	A	**91.7**	Yes	NS3	1369	R1405Q
**4931**	4942	5038	C	T	**99.4**	no	NS3		
**5222**	5233	5329	T	G	**9.9**	no	NS3		
**5272**	5283	5379	A	T	**99.8**	Yes	NS3	1644	Y1680F
**5336**	5347	5443	A	G	**10.8**	no	NS3		
**5357**	5368	5464	A	G	**55.0**	no	NS3		
**5381**	5392	5488	T	C	**16.4**	no	NS4A		
**5385**	5396	5492	A	G	**10.1**	Yes	NS4A	1682	I1718V
**5434**	5445	5541	A	G	**41.1**	Yes	NS4A	1698	K1734R
**5481**	5492	5588	A	G	**22.0**	Yes	NS4B	1714	R1750G
**5489**	5500	5596	T	C	**35.1**	no	NS4B		
**5495**	5506	5602	C	T	**35.0**	no	NS4B		
**5523**	5534	5630	T	C	**23.7**	no	NS4B		

**5600**	5611	5707	A	G	**22.5**	no	NS4B		
**5672**	5683	5779	A	G	**7.6**	no	NS4B		
**5738**	5749	5845	T	C	**20.5**	no	NS4B		
**5795**	5806	5902	C	T	**18.7**	no	NS4B		
**5865**	5876	5972	A	G	**33.9**	Yes	NS4B	1842	I1878V
**6147**	6158	6254	A	G	**36.6**	Yes	NS4B	1936	T1972A
**6365**	6376	6472	C	T	**26.6**	no	NS5A		
**6401**	6412	6508	C	A	**5.1**	no	NS5A		
**6455**	6466	6562	T	C	**48.5**	no	NS5A		
**6458**	6469	6565	C	A	**5.1**	no	NS5A		
**6521**	6532	6628	T	C	**29.6**	no	NS5A		
**6552**	6563	6659	G	T	**6.5**	Yes	NS5A	2071	A2107S
**6559**	6570	6666	A	G	**9.1**	Yes	NS5A	2073	K2109R
**6625**	6636	6732	A	G	**29.8**	Yes	NS5A	2095	Q2131R
**6647**	6658	6754	A	G	**29.7**	no	NS5A		
**6993**	6992	7088	A	G	**9.3**	Yes	NS5A	2218	T2250A
**7074**	7073	7169	G	A	**5.7**	Yes	NS5A	2245	E2277K
**7082**	7081	7177	G	T	**9.4**	no	NS5A		
**7084**	7083	7179	C	A	**14.9**	Yes	NS5A	2248	S2280Y
**7102**	7101	7197	A	G	**10.2**	Yes	NS5A	2254	D2286G
**7108**	7107	7203	T	C	**31.7**	Yes	NS5A	2256	L2288P
**7111**	7110	7206	A	C	**17.5**	Yes	NS5A	2257	E2289A
**7161**	7160	7256	T	C	**99.9**	Yes	NS5A	2274	C2306R
**7208**	7207	7303	A	T	**18.4**	no	NS5A		
**7426**	7425	7521	G	A	**13.6**	Yes	NS5A	2362	G2394D
**7433**	7444	7540	C	T	**20.4**	no	NS5A		

**7442**	7453	7549	A	G	**99.8**	no	NS5A		
**7464**	7475	7571	G	A	**22.6**	Yes	NS5A	2375	G2411S
**7535**	7600	7696	A	G	**5.9**	no	NS5A		
**7553**	7618	7714	G	A	**8.1**	no	NS5A		
**7565**	7630	7726	T	C	**23.7**	no	NS5A		
**7593**	7658	7754	G	A	**78.7**	Yes	NS5A	2418	V2472M
**7662**	7727	7823	T	C	**6.0**	no	NS5B		
**7777**	7842	7938	T	C	**11.0**	Yes	NS5B	2479	V2533A
**7877**	7942	8038	T	C	**21.0**	no	NS5B		
**8136**	8201	8297	A	G	**29.9**	Yes	NS5B	2599	T2653A
**8144**	8209	8305	G	T	**37.4**	no	NS5B		
**8150**	8215	8311	T	C	**5.0**	no	NS5B		
**8198**	8263	8359	A	G	**52.4**	no	NS5B		
**8245**	8310	8406	T	C	**52.1**	Yes	NS5B	2635	M2689T
**8693**	8758	8854	A	G	**9.4**	no	NS5B		
**8777**	8842	8938	C	T	**99.1**	no	NS5B		
**8819**	8884	8980	T	C	**29.8**	no	NS5B		
**8828**	8893	8989	G	A	**9.6**	no	NS5B		
**8897**	8962	9058	T	A	**99.9**	no	NS5B		
**8949**	9014	9110	T	G	**50.7**	Yes	NS5B	2870	S2924A
**9097**	9162	9258	T	C	**5.1**	Yes	NS5B	2919	V2973A
**9152**	9217	9313	A	G	**13.7**	no	NS5B		
**9212**	9277	9373	T	C	**27.1**	no	NS5B		
**9299**	9364	9460	C	T	**43.8**	no	NS5B		

**p20Hpop ref to H77**	**ref to Jc1**	**ref to Jc1-SP**	**Reference**	**Allele**	**Frequency**	**Coding**	**Coding region**	**aa(H77)**	**aa (Jc1sp)**
29	28	28	G	A	**99.6**	no	5UTR		
**215**	214	214	A	G	**6.1**	no	5UTR		
**302**	301	301	T	C	**99.8**	no	5UTR		
**341**	340	340	C	T	**98.5**	no	5UTR		
**384**	383	383	A	T	**98.1**	Yes	Core	15	T15S
**394**	393	393	G	A	**5.2**	Yes	Core	18	R18H
**402**	401	401	G	A	**46.0**	Yes	Core	21	D21N
**407**	406	406	T	C	**7.9**	no	Core		
**521**	520	520	A	G	**10.4**	no	Core		
**581**	580	580	A	G	**12.8**	no	Core		
**665**	664	664	C	T	**7.1**	no	Core		
**670**	669	669	A	G	**57.9**	Yes	Core	110	N110S
**680**	679	679	G	A	**5.9**	no	Core		
**911**	910	910	C	A	**62.9**	no	Core		
**923**	922	922	G	A	**35.3**	no	E1		
**1025**	1024	1024	G	C	**9.2**	no	E1		
**1082**	1081	1081	C	T	**39.5**	No	E1		
**1212**	1211	1211	T	A	**97.7**	Yes	E1	291	F291I
**1259**	1258	1258	C	T	**16.8**	no	E1		
**1274**	1273	1273	T	A	**38.9**	no	E1		
**1341**	1340	1340	T	C	**5.8**	no	E1		
**1358**	1357	1357	T	C	**98.2**	no	E1		
**1451**	1450	1450	A	G	**98.1**	no	E1		
**1453**	1452	1452	T	C	**23.0**	Yes	E1	371	V371A

**1488**	1487	1523	G	A	**41.0**	Yes	E2	383	A395T
**1536**	1535	1535	A	G	**97.9**	Yes	E2	399	T399A
**1560**	1559	1559	A	T	**57.3**	Yes	E2	407	T407S
**1604**	1603	1603	G	A	**5.3**	no	E2		
**1626**	1625	1625	A	G	**99.8**	Yes	E2	429	N429D
**1760**	1759	1795	A	C	**42.1**	Yes	E2	473	Q485H
**1802**	1801	1837	T	C	**42.6**	no	E2		
**1817**	1816	1852	A	G	**8.0**	Yes	E2	488	C500R
**2115**	2126	2162	A	G	**96.3**	no	E2		
**2178**	2189	2225	T	C	**15.2**	Yes	E2	592	T608A
**2229**	2240	2276	A	C	**56.1**	Yes	E2	613	Y629H
**2249**	2260	2296	T	C	**13.5**	no	E2		
**2318**	2329	2365	A	G	**96.7**	no	E2		
**2369**	2380	2416	T	C	**6.5**	no	E2		
**2378**	2389	2425	C	T	**55.7**	no	E2		
**2471**	2482	2518	A	G	**96.5**	no	E2		
**2507**	2518	2554	A	G	**43.7**	no	E2		
**2628**	2639	2675	A	G	**99.8**	no	E2		
**2686**	2697	2733	T	C	**8.1**	Yes	p7	763	N779D
**2696**	2707	2743	A	G	**96.4**	Yes	p7	782	V798A
**2784**	2795	2831	T	G	**15.1**	no	P7		
		2859	T	C	**96.7**	Yes	Sig Pep		F831V
		2868	A	G	**56.0**	Yes	Sig Pep		V840A
		2880	A	G	**96.7**	Yes	Sig Pep		L843S
		2887	T	C	**45.2**	Yes	Sig Pep		D847G
**2792**	2803	2899	G	C	**45.6**	no	NS2		

**2819**	2830	2926	C	T	**98.1**	Yes	NS2	818	G853H
**2848**	2859	2955	A	G	**96.8**	no	NS2		
**2970**	2981	3077	G	T	**96.4**	Yes	NS2	836	K872R
**2990**	3001	3097	T	C	**96.2**	Yes	NS2	877	V913F
**3003**	3014	3110	A	C	**99.6**	no	NS2		
**3029**	3040	3136	T	G	**99.6**	Yes	NS2	888	I924L
**3083**	3094	3190	A	G	**53.1**	no	NS2		
**3108**	3119	3215	G	A	**96.6**	no	NS2		
**3162**	3164	3260	C	T	**5.1**	Yes	NS2	923	A959T
**3287**	3298	3394	G	A	**42.9**	no	NS2		
**3341**	3352	3448	T	C	**95.1**	no	NS2		
**3422**	3433	3529	T	A	**6.8**	no	NS2		
**3437**	3448	3544	T	C	**6.0**	no	NS3		
**3539**	3550	3646	T	G	**5.2**	no	NS3		
**3647**	3658	3754	G	C	**5.4**	no	NS3		
**3679**	3690	3786	G	C	**92.7**	no	NS3		
**3731**	3742	3838	A	G	**41.9**	Yes	NS3	1113	S1149T
**3746**	3757	3853	G	A	**39.8**	no	NS3		
**3755**	3766	3862	T	C	**6.3**	no	NS3		
**3776**	3787	3883	C	T	**6.5**	no	NS3		
**3899**	3910	4006	T	C	**20.7**	no	NS3		
**3911**	3922	4018	C	T	**25.9**	no	NS3		
**4031**	4042	4138	T	C	**9.2**	no	NS3		
**4196**	4207	4303	G	T	**94.8**	no	NS3		
**4266**	4277	4373	G	T	**94.6**	no	NS3		
**4328**	4339	4435	C	A	**94.4**	Yes	NS3	1309	A1345S

**4382**	4393	4489	T	C	**17.1**	no	NS3		
**4391**	4402	4498	A	T	**42.3**	no	NS3		
**4430**	4441	4537	A	C	**5.5**	no	NS3		
**4447**	4458	4554	G	A	**95.6**	no	NS3		
**4754**	4765	4861	T	A	**40.3**	Yes	NS3	1369	R1405Q
**4994**	5005	5101	T	C	**48.4**	no	NS3		
**5127**	5138	5234	A	C	**45.2**	no	NS3		
**5183**	5194	5290	C	T	**77.9**	no	NS3		
**5255**	5266	5362	A	G	**97.8**	no	NS3		
**5272**	5283	5379	A	T	**99.9**	no	NS3		
**5294**	5305	5401	T	C	**13.7**	Yes	NS3	1644	Y1680F
**5345**	5356	5452	A	G	**8.5**	no	NS3		
**5357**	5368	5464	A	G	**97.9**	no	NS3		
**5363**	5374	5470	C	T	**97.4**	no	NS3	1687	H1723R
**5381**	5392	5488	T	C	**10.9**	no	NS3		
**5401**	5412	5508	A	G	**97.7**	Yes	NS3	1681	R1750G
**5481**	5492	5588	A	G	**45.5**	Yes	NS4A	1687	Q1723R
**5501**	5512	5608	G	A	**96.5**	no	NS4B		
**5544**	5555	5651	T	C	**15.8**	no	NS4B		
**5582**	5593	5689	A	G	**97.1**	no	NS4B		
**5807**	5818	5914	A	G	**97.1**	no	NS4B		
**5825**	5836	5932	C	A	**11.9**	no	NS4B		
**6020**	6031	6127	G	A	**98.1**	no	NS4B		
**6098**	6109	6205	T	C	**46.8**	no	NS4B		
**6218**	6229	6325	A	G	**8.1**	no	NS4B		
**6293**	6304	6400	T	C	**14.2**	no	NS4B		

**6299**	6310	6406	C	T	**12.9**	no	NS5A		
**6332**	6343	6439	T	C	**98.1**	no	NS5A		
**6353**	6364	6460	C	A	**98.4**	no	NS5A		
**6392**	6403	6499	T	C	**46.4**	no	NS5A		
**6434**	6445	6541	C	T	**51.5**	no	NS5A		
**6440**	6451	6547	C	T	**57.9**	no	NS5A		
**6455**	6466	6562	T	C	**98.5**	no	NS5A		
**6524**	6535	6631	T	C	**8.5**	no	NS5A		
**6560**	6571	6667	A	G	**95.5**	no	NS5A		
**6567**	6578	6674	A	G	**46.7**	no	NS5A		
**6792**	6803	6899	C	A	**31.9**	Yes	NS5A	2076	T2112A
**6982**	6993	7089	C	T	**5.6**	Yes	NS5A	2151	L2187I
**6997**	7008	7104	G	T	**5.0**	Yes	NS5A	2214	T2250I
**7055**	7066	7162	T	C	**14.3**	Yes	NS5A	2219	S2250I
**7063**	7074	7170	A	G	**12.0**	no	NS5A		
**7084**	7095	7191	T	C	**15.3**	Yes	NS5A	2241	E2277G
**7149**	7160	7256	T	C	**99.8**	Yes	NS5A	2248	V2284A
**7170**	7181	7277	T	C	**8.8**	Yes	NS5A	2270	C2306R
**7189**	7200	7296	C	T	**23.5**	Yes	NS5A	2277	F2313L
**7287**	7298	7394	A	G	**35.2**	Yes	NS5A	2283	A2318V
**7290**	7301	7397	A	G	**24.5**	Yes	NS5A	2316	K2352E
**7442**	7453	7549	A	C	**98.5**	Yes	NS5A	2317	K2353E
**7467**	7478	7574	G	C	**36.3**	no	NS5A		
**7468**	7479	7575	A	G	**61.3**	Yes	NS5A	2376	E2412Q
**7479**	7490	7586	T	G	**6.5**	Yes	NS5A	2376	E2412G
**7479**	7490	7586	T	C	**7.1**	Yes	NS5A	2380	S2416A

**7505**	7516	7612	G	C	**25.3**	Yes	NS5A	2380	S2416P
**7576**	7587	7683	A	T	**13.3**	Yes	NS5A	2389	M2424I
**7593**	7658	7754	G	A	**97.4**	Yes	NS5A	2412	Q2448L
**7649**	7714	7810	C	T	**19.4**	Yes	NS5A	2418	V2472M
**7709**	7774	7870	G	A	**43.9**	no	NS5B		
**7727**	7792	7888	A	G	**54.4**	no	NS5B		

Mad18pop ref to H77	ref to Jc1	ref to Jc1-SP	Reference	Allele	Frequency	Coding	Coding region	aa(H77)	aa (Jc1sp)
**29**	28	28	G	A	**98.7**	no	5UTR		
**302**	301	301	T	C	**99.4**	no	5UTR		
**324**	323	323	T	C	**99.2**	no	5UTR		
**341**	340	340	C	T	**99.9**	no	5UTR		
**384**	383	383	A	T	**99.7**	Yes	Core	15	T15S
**643**	642	642	G	A	**99.6**	Yes	Core	101	R101Q
**740**	739	739	C	T	**99.7**	no	Core		
**771**	770	770	C	T	**6.5**	Yes	Core	144	L144F
**858**	857	857	T	C	**99.8**	Yes	Core	173	S173P
**860**	859	859	C	T	**81.5**	no	Core		
**869**	868	868	C	T	**99.7**	no	Core		
**923**	922	922	G	A	**99.2**	no	E1		
**995**	994	994	T	A	**99.0**	no	E1		
**1004**	1003	1003	C	T	**13.2**	no	E1		
**1082**	1081	1081	C	T	**99.3**	no	E1		
**1212**	1211	1211	T	A	**98.9**	Yes	E1	291	F291I
**1259**	1258	1258	C	T	**99.2**	no	E1		
**1274**	1273	1273	T	A	**99.1**	no	E1		
**1358**	1357	1357	T	C	**99.7**	no	E1		
**1451**	1450	1450	A	G	**99.9**	no	E1		
**1482**	1481	1517	A	G	**6.9**	Yes	Flag tag		D390N
**1488**	1487	1523	G	A	**99.8**	Yes	E2	383	A395T
**1500**	1499	1535	A	G	**100.0**	Yes	E2	387	T399A

**1590**	1589	1625	A	G	**99.9**	Yes	E2	417	N429D
**1760**	1759	1795	A	C	**99.7**	Yes	E2	473	Q485H
**2115**	2126	2162	A	G	**99.7**	Yes	E2	592	T608A
**2177**	2188	2224	C	T	**99.6**	no	E2		
**2198**	2209	2245	C	T	**98.3**	no	E2		
**2318**	2329	2365	A	G	**99.9**	no	E2		
**2354**	2365	2401	A	G	**99.9**	no	E2		
**2459**	2470	2506	A	C	**99.8**	no	E2		
**2471**	2482	2518	A	G	**99.6**	no	E2		
**2628**	2639	2675	A	G	**99.9**	Yes	P7	763	N779D
**2649**	2660	2696	A	G	**5.9**	Yes	P7	770	I786V
**2696**	2707	2743	A	G	**99.7**	no	P7		
	2732	2828	A	G	**99.9**	Yes	Sig Pep		N830D
	2763	2859	T	C	**99.9**	Yes	Sig Pep		V840A
	2784	2880	A	G	**99.9**	Yes	Sig Pep		D847G
**2819**	2830	2926	C	T	**99.7**	no	NS2		
**2848**	2859	2955	A	G	**99.7**	Yes	NS2	836	K872R
**2885**	2896	2992	T	C	**99.8**	no	NS2		
**2960**	2971	3067	C	T	**99.8**	no	NS2		
**2970**	2981	3077	G	T	**99.8**	Yes	NS2	877	V913F
**2978**	2989	3085	A	G	**15.2**	no	NS2		
**2990**	3001	3097	T	C	**98.3**	no	NS2		
**3003**	3014	3110	A	C	**99.8**	Yes	NS2	888	I924L
**3029**	3040	3136	T	G	**99.7**	no	NS2		
**3108**	3119	3215	G	A	**99.6**	Yes	NS2	923	A959T
**3287**	3298	3394	G	A	**98.9**	no	NS2		

**3330**	3341	3437	C	A	**99.8**	Yes	NS2	997	L1033I
**3341**	3352	3448	T	C	**99.9**	no	NS2		
**3389**	3400	3496	T	C	**99.4**	no	NS2		
**3679**	3690	3786	G	C	**99.5**	Yes	NS3	1113	S1149T
**3731**	3742	3838	A	G	**99.9**	no	NS3		
**3899**	3910	4006	T	C	**99.8**	no	NS3		
**4196**	4207	4303	G	T	**99.7**	no	NS3		
**4266**	4277	4373	G	T	**99.8**	Yes	NS3	1309	A1345S
**4328**	4339	4435	C	A	**99.9**	no	NS3		
**4334**	4345	4441	G	C	**99.9**	no	NS3		
**4382**	4393	4489	T	C	**99.8**	no	NS3		
**4391**	4402	4498	A	T	**98.8**	no	NS3		
**4447**	4458	4554	G	A	**99.9**	Yes	NS3	1369	R1405Q
**4457**	4468	4564	G	A	**70.0**	no	NS3		
**4931**	4942	5038	C	T	**99.2**	no	NS3		
**5127**	5138	5234	A	C	**99.9**	no	NS3		
**5183**	5194	5290	C	T	**99.6**	no	NS3		
**5195**	5206	5302	T	G	**99.9**	no	NS3		
**5255**	5266	5362	A	G	**98.9**	no	NS3		
**5272**	5283	5379	A	T	**99.9**	Yes	NS3	1644	Y1680F
**5294**	5305	5401	T	C	**99.5**	no	NS3		
**5357**	5368	5464	A	G	**99.8**	no	NS3		
**5363**	5374	5470	C	T	**99.4**	no	NS3		
**5401**	5412	5508	A	G	**99.9**	Yes	NS4A	1687	H1723R
**5501**	5512	5608	G	A	**97.3**	no	NS4B		
**5544**	5555	5651	T	C	**99.9**	no	NS4B		

**5582**	5593	5689	A	G	**99.9**	no	NS4B		
**5669**	5680	5776	A	G	**5.7**	no	NS4B		
**5807**	5818	5914	A	G	**98.9**	no	NS4B		
**5837**	5848	5944	T	C	**99.9**	no	NS4B		
**6020**	6031	6127	G	A	**99.1**	no	NS4B		
**6273**	6284	6380	C	T	**5.9**	Yes	NS5A	1978	R2014C
**6332**	6343	6439	T	C	**97.3**	no	NS5A		
**6347**	6358	6454	G	A	**5.4**	no	NS5A		
**6353**	6364	6460	C	A	**99.9**	no	NS5A		
**6455**	6466	6562	T	C	**99.9**	no	NS5A		
**6560**	6571	6667	A	G	**98.3**	no	NS5A		
**6567**	6578	6674	A	G	**99.2**	Yes	NS5A	2076	T2112A
**7064**	7063	7159	G	C	**67.2**	no	NS5A		
**7161**	7160	7256	T	C	**99.8**	Yes	NS5A	2274	C2306R
**7201**	7200	7296	C	T	**99.7**	Yes	NS5A	2287	A2319V
**7320**	7319	7415	C	T	**98.6**	Yes	NS5A	2327	P2359S
**7361**	7360	7456	A	G	**99.7**	Yes	NS5A	2341	I2372M
**7442**	7453	7549	A	C	**99.6**	Yes	NS5A	2368	E2403D
**7468**	7479	7575	A	G	**99.8**	Yes	NS5A	2376	E2412G
**7522**	7587	7683	A	T	**65.5**	Yes	NS5A	2394	Q2448L
**7534**	7599	7695	T	C	**99.7**	Yes	NS5A	2398	V2452A
**7593**	7658	7754	G	A	**99.9**	Yes	NS5A	2418	V2472M
**7727**	7792	7888	A	G	**99.9**	no	NS5B		
**7775**	7840	7936	A	G	**98.9**	no	NS5B		
**7934**	7999	8095	G	A	**99.8**	no	NS5B		
**8198**	8263	8359	A	G	**99.9**	no	NS5B		

**8245**	8310	8406	T	C	**99.9**	Yes	NS5B	2635	M2689T
**8339**	8404	8500	C	T	**99.4**	no	NS5B		
**8777**	8842	8938	C	T	**99.2**	no	NS5B		
**8897**	8962	9058	T	A	**99.9**	no	NS5B		
**8949**	9014	9110	T	G	**99.9**	Yes	NS5B	2870	S2924A
**9020**	9085	9181	T	C	**99.4**	no	NS5B		
**9131**	9196	9292	C	G	**99.9**	no	NS5B		
**9212**	9277	9373	T	C	**99.7**	no	NS5B		
**9575**	9640	9736	T	C	**15.5**	no	3UTR		
**9576**	9641	9737	G	A	**9.2**	no	3UTR		

Table S2. IC50 and IC90 values from HCV neutralization or host factor restriction.

**Virus**	**HCV E1E2(Fab)**		**SRB1**		**CD81(JS-81)**		**CD81(5A1)**	
	**IC50 (µg/ml)**	**IC90 (µg/ml)**	**IC50 (µ/ml)**	**IC90 (µg/ml)**	**IC50 (µg/ml)**	**IC90 (µg/ml)**	**IC50 (µg/ml)**	**IC90 (µg/ml)**
**Jc1-WT**	>10	>10	0.30(±0.11)	>10	0.19(±0.15)	0.40(±0.05)	0.52 (±0.24)	1.86(±0.49)
**Mad18cl**	0.37(±0.41)	3.01(±3.00)	>10	>10	0.09(±0.02)	1.59(±0.38)	0.41(±0.36)	14.71(±15.46)
**M18WTE1E2**	>10	>10	0.85(±1.35)	>10	0.08(±0.01)	0.23(±0.06)	0.26(±0.20)	1.25(±0.60)
**P100cl**	0.02(±0.01)	0.128(±0.07)	>10	>10	0.23(±0.12)	0.87(±0.48)	1.48(±0.23)	11.98(±7.03)
**P100WTE1E2**	>10	>10	0.24(±0.13)	>10	0.06(±0.02)	0.48(±0.16)	1.07(±0.21)	5.17(±0.41)
**Jc1-mCD81**	0.016(±0.01)	0.50(±0.75)	>10	>10	0.13(±0.14)	1.13(±1.18)	0.33(±0.20)	7.15(±6.53)
