# Supplementary material for: Adapted hepatitis C virus clone infects innate immunity-deficient mouse hepatocytes with minimal human HCV entry factors
Source: JHEP Rep. 2025 Jan 18;7(5):101328. doi: 10.1016/j.jhepr.2025.101328 (PMC11999267; doi:10.1016/j.jhepr.2025.101328)
Supplement: Multimedia component 2 [file mmc2.docx]

**Journal of Hepatology**

**CTAT methods**

Tables for a “Complete, Transparent, Accurate and Timely account” (CTAT) are now mandatory for all revised submissions. The aim is to enhance the reproducibility of methods.

- Only include the parts relevant to your study
- Refer to the CTAT in the main text as ‘Supplementary CTAT Table’
- Do not add subheadings
- Add as many rows as needed to include all information
- Only include one item per row

**If the CTAT form is not relevant to your study, please outline the reasons why:**

|  |
| --- |

- 1. **Antibodies**

| **Name** | **Citation** | **Supplier** | **Cat no.** | **Clone no.** |
| --- | --- | --- | --- | --- |
| HCV NS5a |  | Cell essentials/Charles Rice, Rockefeller University |  | 9E10 |
| mouse anti-HCV Core clone |  | Jean Dubuisson, Lille University |  | C7-50 |
| rabbit anti-GAPDH |  | Sigma-Aldrich | #G9545 |  |
| Goat anti-rabbit HRP IgG |  | Jackson | #111035003 |  |
| Alexa Fluor 488 rabbit anti-mouse immunoglobulin G |  | Thermo Fisher | A32723 |  |
| Goat anti-mouse HRP IgG |  | Sigma-Aldrich | # A4416 |  |
| anti-CD81 |  | BD Pharmingen | 555675 | JS-81 |
| anti-CD81 |  | SCBT | #SC28962 | 5A6 |
| anti-SR-BI |  | Phillip Mueleman, Gent University |  | C16-71 |
| FAB mix AR3C/HC84.1 |  | Thomas Krey, University Luebeck |  |  |

- 1. **Cell lines**

| **Name** | **Citation** | **Supplier** | **Cat no.** | **Passage no.** | **Authentication test method** |
| --- | --- | --- | --- | --- | --- |
| Huh-7.5 | Blight KJ, McKeating JA, Rice CM. 2002. J Virol 76:13001-14. |  |  | 25-35 |  |
| MLT-5H | Frentzen A, Anggakusuma, Gurlevik E, et al. 2014.  . Hepatology 59:78-88. |  |  | 5-15 |  |
| Lunet N#3 hCD81 | Bitzegeio J, Bankwitz D, Hueging K, et al. 2010.  . PLoS Pathog  6:e1000978. |  |  | 15-55 |  |
| Lunet N#3 mCD81 | Bitzegeio J, Bankwitz D, Hueging K, et al. 2010.  . PLoS Pathog  6:e1000978.. |  |  | 15-55 |  |

- 1. **Organisms**

| **Name** | **Citation** | **Supplier** | **Strain** | **Sex** | **Age** | **Overall n number** |
| --- | --- | --- | --- | --- | --- | --- |
|  |  |  |  |  |  |  |

- 1. **Sequence based reagents**

| **Name** | **Sequence** | **Supplier** |
| --- | --- | --- |
|  |  |  |

- 1. **Biological samples**

| **Description** | **Source** | **Identifier** |
| --- | --- | --- |
| Primary human hepatocytes | Hannover Medical school (MHH) |  |
| Primary mouse hepatocytes | Twincore |  |
| Primary Macaque hepatocytes | Lonza |  |

- 1. **Deposited data**

| **Name of repository** | **Identifier** | **Link** |
| --- | --- | --- |
|  |  |  |

- 1. **Software**

| **Software name** | **Manufacturer** | **Version** |
| --- | --- | --- |
| CLC Genomics Workbench | Qiagen | V23 |
| Mega | Mega software | V6 |
| SnapGene V7.02 | dotmatics | V7.02 |
| Graphpad | Prism | V9 |

- 1. **Other (e.g. drugs, proteins, vectors etc.)**

| **Reagent** | **Supplier** | **Cat. No** |
| --- | --- | --- |
| Teleprevir | **Selleckchem** | **S1538** |
| Ruxolitinib | **Adipogen** | **AG-CR1-3624** |

- 1. **Please provide the details of the corresponding methods author for the manuscript:**

| Professor Thomas Pietschmann  Institute of Experimental Virology  Twincore  Feodor Lynen str 7-9  30625 Hannover  Tel: + (0) 511 – 22 00 27 – 130/131  Fax: + (0) 511 – 22 00 27 – 139  Thomas Pietschmann@twincore.de |
| --- |

**2.0 Please confirm for randomised controlled trials all versions of the clinical protocol are included in the submission. These will be published online as supplementary information.**

|  |
| --- |
